# Structural Features Dictate the Photoelectrochemical Activities of Two-Dimensional MoSe_2_ and WSe_2_ Nanostructures

**DOI:** 10.1021/acs.jpcc.1c01265

**Published:** 2021-03-31

**Authors:** Péter S. Tóth, Gábor Szabó, Csaba Janáky

**Affiliations:** † MTA Premium Post Doctorate Research Program, University of Szeged, Szeged 6720, Hungary; ‡ Department of Physical Chemistry and Materials Science, Interdisciplinary Excellence Center, University of Szeged, Szeged 6720, Hungary

## Abstract

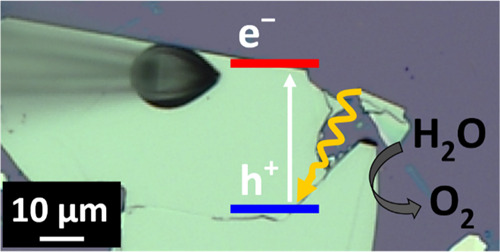

The exfoliation of layered materials into two-dimensional (2D) semiconductors creates new structural domains, for example, basal planes, defect-rich in-planes, and edge sites. These surface species affect the photoelectrochemical (PEC) performance, which in turn determines their applicability in solar energy conversion technologies. In this study, a custom-designed microdroplet cell-based spatially resolved PEC approach was employed to identify the structural parts and to measure the PEC activity of the mechanically exfoliated MoSe_2_ and WSe_2_ nanosheets for bulk, few-layer, and monolayer specimens. The PEC performance decreased with the decreasing thickness of nanoflakes, and the relative PEC activity (photo/total current) reduced by introducing more defects to the 2D flakes: 1–3% loss was found for in-plane defects and 30–40% for edge defects. While edge sites act as charge carrier recombination centers, their electrocatalytic activity is higher than that of the basal planes. The comparison of PEC activity of micromechanically and liquid phase exfoliated bulk and few-layer MoSe_2_ and WSe_2_ flakes further confirmed that the PEC performance of 2D flakes decreases with an increasing number of edge sites.

## Introduction

Among inorganic layered species, transition metal dichalcogenides (TMDCs) have become attractive candidates in search for new materials for nanoelectronics and catalysis. In such applications, their appropriate band gap energy, high chemical stability, and good electrocatalytic properties can be harnessed.^1^ The development of two-dimensional (2D) material-based technologies further accelerated the research activity on 2D crystals beyond graphene.^2^ The exfoliation of bulk crystals to nanoflakes results in a high surface-to-volume ratio, enabling electrochemical application. Furthermore, exfoliation leads to volume, mass, and the cost reduction of electrochemical devices, such as supercapacitors, batteries, and hydrogen storage units.^3^ The solid understanding of the electrochemical (EC) and photoelectrochemical (PEC) behavior of these 2D semiconductors will allow us to fully harness the as-elucidated properties, ultimately leading to application in catalysis and energy conversion. The role of defects has been long recognized in the electrochemistry of TMDCs. The activity of the basal- (smooth) and defect-rich (stepped) plane was investigated in the 1980s and 1990s already, focusing on bulk single crystals.^4−7^ It was proposed that defects and edge sites attract adsorbates, which create surface states within the band gap, acting as recombination centers for the photogenerated charge carriers. These surface states also increase the photogeneration of charge carriers by photons with lower energy compared to the band gap (i.e., midgap states).^8^ The defect-rich plane has higher EC and lower PEC activity than the basal plane. These states also behave as recombination centers for the photogenerated charge carriers, which explains the lower PEC activity.^4^

Nanostructured 2D materials gained momentum after the discovery of graphene in 2004 and the first promising results with 2D MoS_2_ catalysts in the hydrogen evolution reaction (HER).^9^ Since then, significant knowledge has accumulated on the effect of basal planes versus edges on the EC properties of monolayer MoS_2_ and their role in the HER.^10−13^ Besides the role of macroscopic defects (steps, terraces, and edges), the importance of atomic defects (atomic vacancies, grain boundaries, or anything where the uniform crystal structure is missing or modified) in catalysis has been also reported.^14−16^ In the case of mechanically exfoliated MoS_2_ and WS_2_ monolayers, atomic-scale defects were created, and the evolution of the Raman and photoluminescence spectra was studied, revealing a distinct defect-related spectral feature in the photoluminescence properties.^17^ Furthermore, it was experimentally verified how the S-vacancies and O-substitution (generated by plasma treatment) result in a highly active catalytic site for HER in the basal plane of MoS_2_ monolayers.^18^ When applying MoS_2_ for CO_2_ electrolysis, the molybdenum-terminated edges were mainly responsible for its catalytic activity.^19^ The general consensus assumes an increased EC activity of the edge (the side of the 2D sheet) in comparison to the basal plane (the smooth, defect-free part of the 2D flake), but reports have been mostly limited to graphite/graphene^20−23^ and MoS_2_.^24−26^

**Figure 1:**
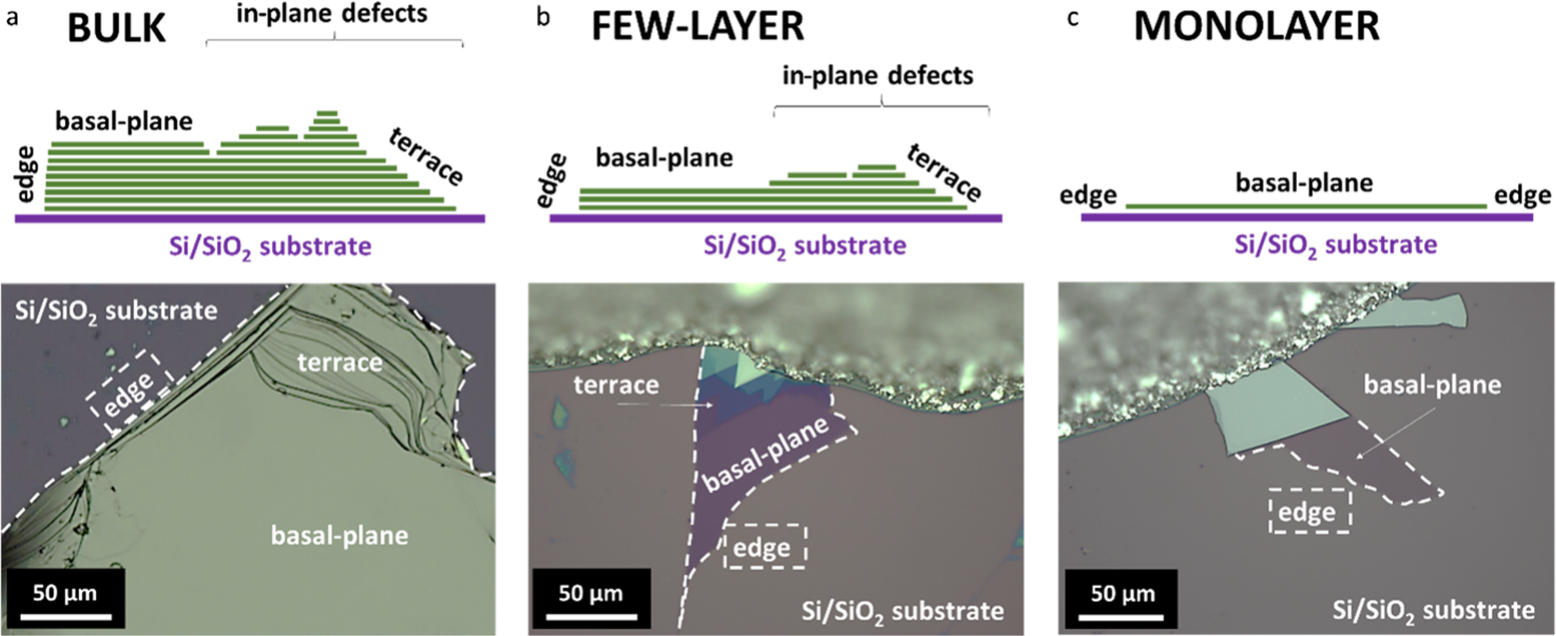
Schemes (top) and optical micrographs (bottom) of different structural domains of MoSe_2_ (a) and WSe_2_ (b, c) nanoflakes in the case of bulk (a), few-layer (b), and monolayer (c) samples. The edge sites are marked with white dashed lines.

Significantly less attention has been devoted to the PEC performance of 2D nanomaterials as a function of their structural properties (see Figure 1, where the different structural specimens are depicted). The electron transfer kinetics on MoS_2_ basal planes was accelerated with the growing number of layers.^27^ The enhanced PEC response of bulk MoS_2_ in comparison to the monolayer was explained by the higher light absorption and the band structure-dependent photogeneration of charge carriers.^4,28,29^ Studies on the spatial variation of the PEC performance of p-type WSe_2_ photocathodes in the reduction of a model redox couple have been reported recently.^30,31^ In situ scanning photocurrent microscopy revealed a variation in the photoconversion efficiency of different structural specimens, including terraces and edge sites.^31^ The photoconversion efficiency decreased on the edge sites mainly because the photogenerated charge carriers recombine. WSe_2_ nanoflakes have been investigated by scanning photocurrent microscopy in a flow electrochemical cell recently.^32^ The internal photon-to-electron conversion efficiency (IPCE) increased with the nanoflake area and decreased at the edges of sheets. Further works examined the effect of Au nanoparticles and laser annealing on the PEC activity of liquid phase exfoliated (LPE) WSe_2_ and MoSe_2_ layers.^33,34^ Even more recently, the PEC activity of TMDCs has been investigated in the function of layer numbers, and other structural domains (defects, terraces, and edges), and compared against the behavior of basal planes using scanning electrochemical microscopy (SECM) and scanning electrochemical cell microscopy (SECCM)^25,26,30,35^ as well as a microdroplet-based approach.^27^ In the case of SECM and SECCM, a laser source is applied as the excitation source. In the early paper of Dryfe’s group,^27^ using the microdroplet approach, white light of the optical microscope was employed to study the PEC performance of MoS_2_ flakes. Although these studies indicated the role of in-plane defects and edges on the EC and PEC properties of 2D TMDCs, the exact nature of these effects is still being challenged.

Considering the practical applications and mass productions of 2D nanomaterials, one of the cheapest methods is the LPE process that also provides high yield. Unfortunately, the PEC activity and energy conversion efficiency of LPE-prepared nanoflakes are typically much lower than those of bulk/single-crystal electrodes.^36,37^ For example, the photocurrents for LPE WSe_2_ nanosheets lie in the few μA cm^–2^ current density range in the absence of the Al_2_O_3_ passivation layer^37^ or without activation with Pt nanoparticles.^36^ Overall, there is a clear need to better understand how the defect nature (in plane defects vs edges) and density affect the PEC activity of 2D semiconductor TMDCs.

In this paper, we report a custom-developed microdroplet-based PEC microscopy approach, which elaborates on the previous Dryfe lab setup^27^ and provides spectral resolution to the spatially resolved PEC studies, as opposed to other similar methodologies. Photovoltammetry and IPCE studies were employed to semiquantitatively compare the PEC behavior of MoSe_2_ and WSe_2_ samples with different layer thicknesses. The systematic PEC study on in-plane defects and edges in bulk, few, and monolayer samples identified the key parameters dictating the PEC performance. Studies in the presence and absence of reversible redox couples allowed us to deconvolute the contribution of charge carrier recombination and charge transfer to the overall PEC activity.

## Experimental Section

### Materials and Methods

Lithium chloride (LiCl, 99.99%) was purchased from Acros Organics. Hexaammineruthenium(III) chloride (Ru(NH_3_)_6_Cl_3_, 98%), potassium chloride (KCl, analytical grade), acetone (≥99.0%), ethanol (≥99.0%), and isopropyl alcohol (IPA, 99.5%) were purchased from Sigma-Aldrich. MoSe_2_ (99.9%, −325 mesh) and WSe_2_ (99.8%, 10–20 μm) powders (Alfa Aesar) were used for liquid phase exfoliation. A fluorine-doped tin oxide-coated glass (FTO, Sigma-Aldrich, surface resistivity ∼7 Ω sq^–1^) was used as a working electrode substrate for LPE prepared samples. Platinum (Pt, >99.99%, 0.2 mm diameter), silver (Ag, 99.99%, 0.14 mm diameter partially exposed PTFE-coated silver wire), and copper (Cu, 99.99%, 0.15 mm diameter) wires were purchased from Advent Research Materials Ltd. All chemicals were used as received, and all solutions were prepared using deionized water (Millipore Direct Q3-UV, 18.2 MΩ cm^–1^).

### Preparation of MoSe_2_ and WSe_2_ Flakes

MoSe_2_ and WSe_2_ single crystals (HQGraphene) were mechanically exfoliated onto insulating oxidized silicon-coated silicon (SiO_2_/Si) wafers (Graphene Supermarket) using the mechanical “Scotch tape” cleavage method (see more details in the Supporting Information). The flakes were then electrically contacted using silver epoxy and a copper wire. The dispersions of liquid phase exfoliated 2D crystals were produced ultrasonically (Elmasonic P70H) and sorted using centrifugation (Hermle, Z366K) by area and thickness to obtain size-selected flakes (see more details in the Supporting Information). The bulk and few-layer flakes containing dispersions were chosen to deposit films onto FTO electrodes using a modified Langmuir–Blodgett method.^38^ Briefly, an Erlenmeyer flask was filled with deionized water, and a few drops of the dispersion were slowly layered on top of water. This film was transferred subsequently onto the FTO glass slide (1.0 cm × 2.0 cm) inserting below the slide using a set of tweezers and pulling off slowly and then repeating the transfer step twice more resulting in a thin film on the FTO surface (81 and 87 μg cm^–2^ loading for bulk and few-layer MoSe_2_ samples, respectively).

### Characterization

The selected ME flakes and structural domains were identified by an optical microscope, and then, morphological and spectroscopic measurements were performed. Raman spectroscopy analysis was carried out with a Senterra II Compact Raman microscope (Bruker) using 532 nm laser excitation wavelength, operating at a power of ≤2.5 mW and a 50× objective. Atomic force microscopy (AFM; NT-MDT Solver AFM microscope) operated in “tapping” mode with a silicon tip on a silicon nitride lever (Nanosensors, Inc., SSS-NCH-type 15 μm long silicon needle with a 10° half cone angle and 2 nm radius of curvature) was also used to analyze samples. The 2D dispersions were characterized morphologically (measuring lateral size) capturing transmission electron microscopy images (TEM; FEI Tecnai G_2_ 20 X-Twin type, operating at an acceleration voltage of 200 kV). Analysis of TEM images was performed using ImageJ software, determining the lateral size of each flake and performing statistical analysis on 100 flakes. The morphology of MoSe_2_ and WSe_2_ films on FTO electrodes was characterized by scanning electron microscopy (SEM; Hitachi S-4700 Type II, operating at 10–15 kV). The absorption spectra of the deposited MoSe_2_/FTO and WSe_2_/FTO films were acquired by an Agilent 8453 UV–visible diode array spectrophotometer in the range of 400–1000 nm.

### Measurements and Data Analysis

The characteristics of AFM and Raman spectroscopy that identified ME flakes, the thickness of specimens, and the structural domains were linked to the optical micrographs. The fraction of the heterogeneous surface covered by defects (θ_defect_) was calculated using AFM height profiles. On a selected area, the vertical dimension was divided by the planar dimension. A Nikon Eclipse LV100ND optical microscope and a DS-Fi3 camera (Nikon Metrology) were used to visualize and select MoSe_2_ flakes where liquid droplets were deposited. The aqueous droplets of both the 6 M LiCl electrolyte and redox mediator containing solution (5 mM Ru(NH_3_)_6_Cl_3_ in 6 M LiCl) were deposited *via* a borosilicate micropipette and a pneumatic microinjector (PV820 Pneumatic PicoPump, WPI) applying argon gas (Ar, Messer, 99.996%). A pair of *ca*. 5 cm long micropipettes was prepared by a P-97 Flaming/Brown micropipette puller (Sutter Instrument) using borosilicate capillary with a filament (outer diameter of 1.5 mm; inner diameter of 1.1 mm, Sutter Instrument). The vertical and horizontal motions of the micropipette sitting on its holder were controlled using a MX7630 micromanipulator and a MC 1000e motion controller (Siskiyou). We aimed to deposit droplets with the size comparable to the studied structural domain. Top view and side view optical images confirmed that the contact area (i.e., the electrode area) is equal to the area calculated from the top view images (5–100 μm in diameter). All electrochemical measurements were controlled by a PGSTAT302N potentiostat (Metrohm Autolab). The microelectrochemical cell was enclosed in a Faraday cage (Figure S1). The potential was measured against the Ag/AgCl reference electrode in 6 M LiCl, which is *ca*. +0.19 V on the SHE scale. The linear sweep and cyclic voltammetric traces were collected at 5 and 50–300 mV s^–1^ scan rates, respectively. The electrochemically active area was illuminated by a halogen fiber-optic light source (Fiber-Lite DC950 Illuminator, 150 W) using either white light or monochromated light using 50 nm steps between 400 and 1000 nm (SPS030 module, KP Technology Ltd.). To illuminate the micron-sized droplets selectively, focusing lenses and fiber-optic cables (50 μm core) were applied (Thorlabs). The power density of illumination (irradiance) was measured using a power sensor (Thorlabs, S302A) and a compact USB power meter with a slim photodiode sensor (Thorlabs, PM16-130). The intensity modulated photocurrent spectroscopy (IMPS) measurement was carried out using the same system, but in this case, the potentiostat was equipped with an FRA32 module (Metrohm-Autolab) and an LED driver kit (Metrohm-Autolab). The IMPS spectra were recorded in the frequency range between 20 kHz and 0.1 Hz using sinusoidal intensity modulation and bias illumination by a white light LED in 6 M LiCl with 5 mM [Ru(NH_3_)_6_]^2+^ solution. The amplitude of the sinusoidal modulation was 10% of the original intensity. Normalization of the measured signal was carried out by determining the number of incident photons employing a Thorlabs power sensor. The IPCE(%) and APCE(%) values were calculated using the following equations
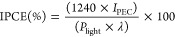

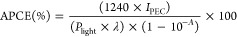
where *I*_PEC_ is the measured photocurrent density, *P*_light_ is the photon flux, λ is the wavelength, and the absorbance is defined as the fraction of electron–hole (e^–^/h^+^) pairs generated by incident photon flux. This absorbance is estimated from the Beer–Lambert law defining the absorbance (*A*) of a sample as the logarithmic ratio of the measured output light intensity (*I*) versus the initial input light intensity (*I*_0_).^39^ All measurements were performed at ambient temperature (23–24 °C). The displayed errors were standard deviations (arithmetic averages of multiple measured values).

## Results and Discussion

**Figure 2:**
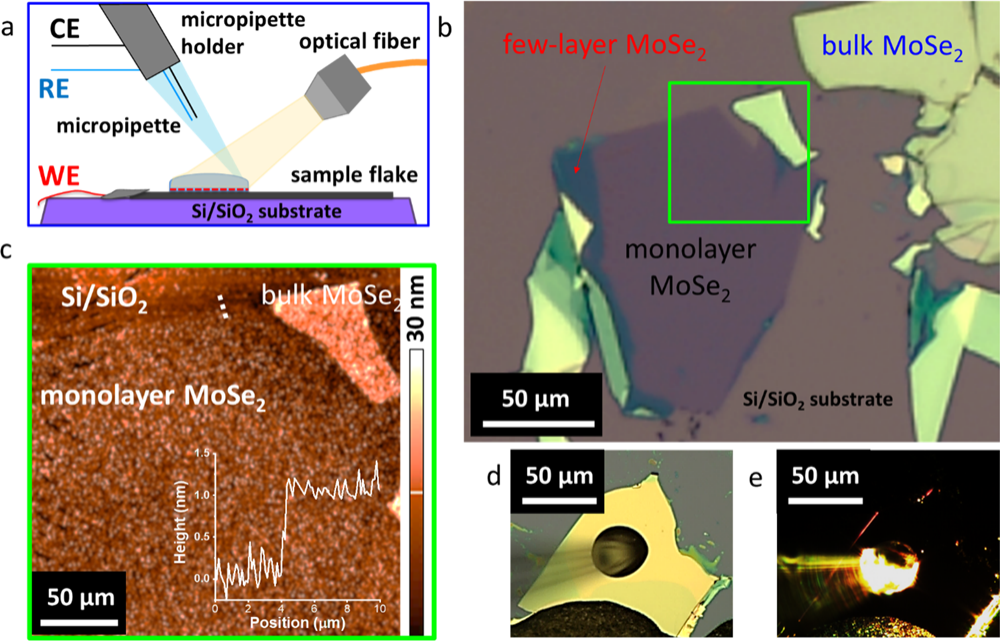
Microscopic characterization of MoSe_2_ flakes with the microdroplet PEC system. (a) Scheme of the PEC setup. (b) Optical micrograph of exfoliated MoSe_2_ flakes on the SiO_2_/Si substrate. (c) AFM image of a selected monolayer/bulk flake region indicated by a light-green square in panel (b). The inset in panel (c) shows the height profile of the monolayer from the region highlighted by the dashed white line. Optical micrographs of a droplet deposited on the surface of a single-crystal bulk MoSe_2_ flake, without (d) and with (e) external white light illumination.

The operation of our custom-designed PEC setup is shown in Figure 2a. First, (i) the sample area is selected under the microscope; then, (ii) a microdroplet is deposited and maintained using a microinjection and manipulator system; subsequently, (iii) the electrochemical experiment is performed with a potentiostat–galvanostat; meanwhile, (iv) the illumination is provided with a fiber-optic light source with controlled wavelength and intensity (see Figure S1 for a more detailed scheme and photograph of the setup). In this three-electrode configuration, the 2D flake area in contact with the microdroplet acts as the working electrode (WE), while the Ag/AgCl pseudoreference electrode (RE) and Pt counter electrode (CE) are embedded in the micropipette tip.^22,23,27^ White or monochromatic light illuminates the droplet cell through a fiber-optic cable on the opposite side of the micropipette. Both the spot size and light intensity were measured before and after each experiment. The mechanically exfoliated (ME) flakes were deposited onto a high optical contrast SiO_2_/Si wafer, allowing the WSe_2_ and MoSe_2_ flakes of all thicknesses to be visualized using optical microscopy (Figure 2b). To confirm the correlation between the contrast on the optical images and the layer thickness, AFM images were recorded (Figure 2c). We selected the flakes and deposited droplets using the light of the microscope *via* the objective (Figure 2d), then this light was turned off, and the EC/PEC measurements were performed by applying external illumination (Figure 2e).

Individual droplets (128) were deposited on different thick MoSe_2_ samples (namely, bulk, few-layer, and monolayer), from which the 5–100 μm diameter size was stable. These droplets were deposited for bulk, few-layer, and monolayer specimens (61, 45, and 22 droplets, respectively). The light spot was focused on the samples, while the intensity was measured as a function of wavelength (Figure S2). At 700 nm, the irradiance power was *ca*. 11 mW cm^–2^. Besides optical microscopy images, AFM and Raman spectroscopy were employed to distinguish among monolayer, few-layer, bulk flakes, and other structural domains (in-plane defects and edges). The thickness of the monolayer flakes was *ca*. 0.9 nm (see the inset of Figure 2c; the incidental adsorbates increased the AFM-derived value^40^). An example of a selected monolayer/bulk region is shown in Figure 2c. The determined thicknesses (obtained from AFM data) for few-layer and bulk flakes lie in the ranges of 2–20 and 65–250 nm, respectively. This gives *ca*. 11 and 171 nm average values to represent the thickness of few-layer and bulk samples. Additional AFM micrographs and height profiles from cross sections of MoSe_2_ and WSe_2_ flakes are presented in Figures S3 and S4. The most intense Raman phonon modes, the fingerprints of 2D TMDCs, and the underlying Si Raman mode^27^ at 520 cm^–1^ were used to assess the flake thickness and identify the in-plane defects for both materials. The out-of-plane (A_1g_) vibration band lies in the range of 240.5–243 cm^–1^ for MoSe_2_ and 256.0–260.5 cm^–1^ and for WSe_2_. The in-plane (E_2g_) lattice vibrations can be found between 251.5 and 248.5 cm^–1^, from monolayer to bulk specimens for WSe_2_ (see Figures S3 and S4).^40−42^

**Figure 3:**
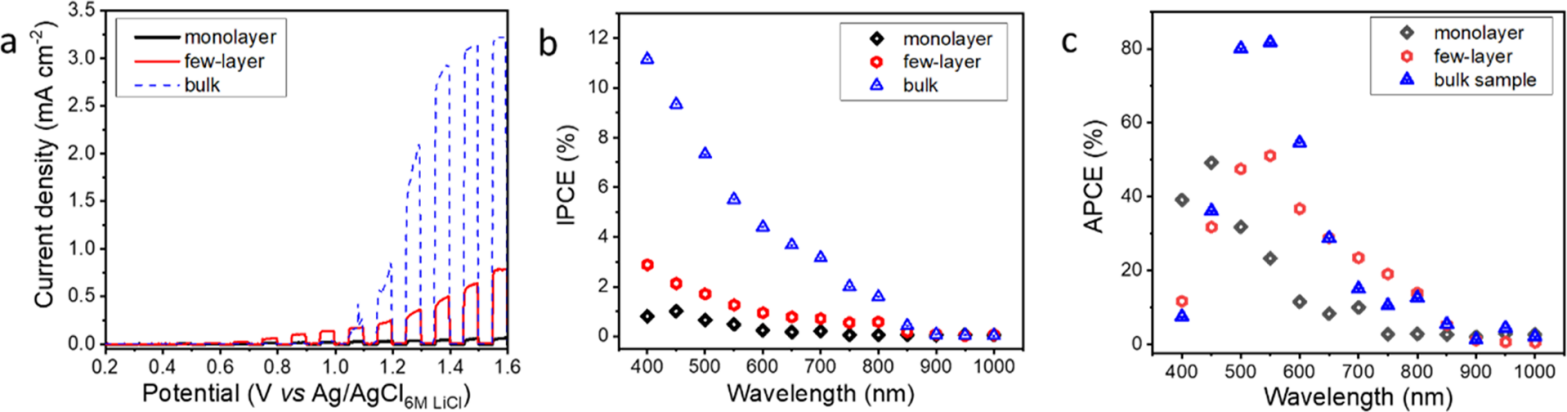
PEC behavior of layered MoSe_2_ specimens (basal planes). (a) LSVs recorded for the illuminated droplets deposited on monolayer, few-layer, and bulk flakes in 6 M LiCl solution; the sweep rate was 5 mV s^–1^. (b) Quantum efficiency curves show different behaviors among the PEC activities of monolayer, few-layer, and bulk flakes. (c) APCE profile, constructed using IPCE data and the estimated absorbance of the flakes.

Using the microscale PEC setup, bulk, few-layer, and monolayer samples of the MoSe_2_ and WSe_2_ nanoflakes were investigated (Figure 1 and Figure S5). The photovoltammograms for the MoSe_2_ specimens are presented in Figure 3a, bearing all the hallmarks of a photoanodic process (i.e., water oxidation in the 6 M LiCl solution). The maximum IPCEs of the few-layer and bulk specimens were achieved at 400 nm, while the monolayer sample reaches the highest efficiency value at 450 nm (Figure 3b). The interlayer coupling and quantum confinement of 2D materials result in a thickness-dependent electronic band structure. With the increasing number of layers (from monolayer to bulk), the band gap energy decreases.^43,44^ The higher photocurrents and larger IPCE values can be simply explained by the much higher light absorption of the few-layer and bulk samples compared to the monolayer.^4,5,28^ Further, photovoltammograms for the WSe_2_ samples are shown in Figure S6.

To deconvolute the obvious difference stemming from the different light absorption of the MoSe_2_ samples with different thicknesses, APCE curves were plotted (Figure 3c). APCE values were calculated using IPCE and estimated absorbance values applying absorption coefficients from the literature and exact thickness on the examples of selected samples of the monolayer (0.9 ± 0.1 nm), few-layer (2.1 ± 0.3 nm), and bulk (26.9 ± 0.7 nm) flakes obtained from AFM measurements (Figure 2 and Figure S7).^45−49^ In this comparison, the differences are much smaller but still remarkable. In the wavelength range between 500 and 600 nm of bulk MoSe_2_, the APCE values exceeded 80%, indicating that almost every absorbed photon yields an extracted electron and hole. In the case of few-layer and monolayer MoSe_2_ flakes, APCE is around 50% between 500 and 600 nm (few-layer) while reaching 30–50% in the range of 450–600 nm (monolayer). These lower carrier collection efficiencies indicate an increased defect density (compared to the bulk sample), which can act as recombination centers.^50^ The band gap energy values of bulk, few-layer, and monolayer MoSe_2_ and WSe_2_ flakes were extracted from the IPCE curves, showing a good match with previously reported values.^48,51,52^

**Figure 4:**
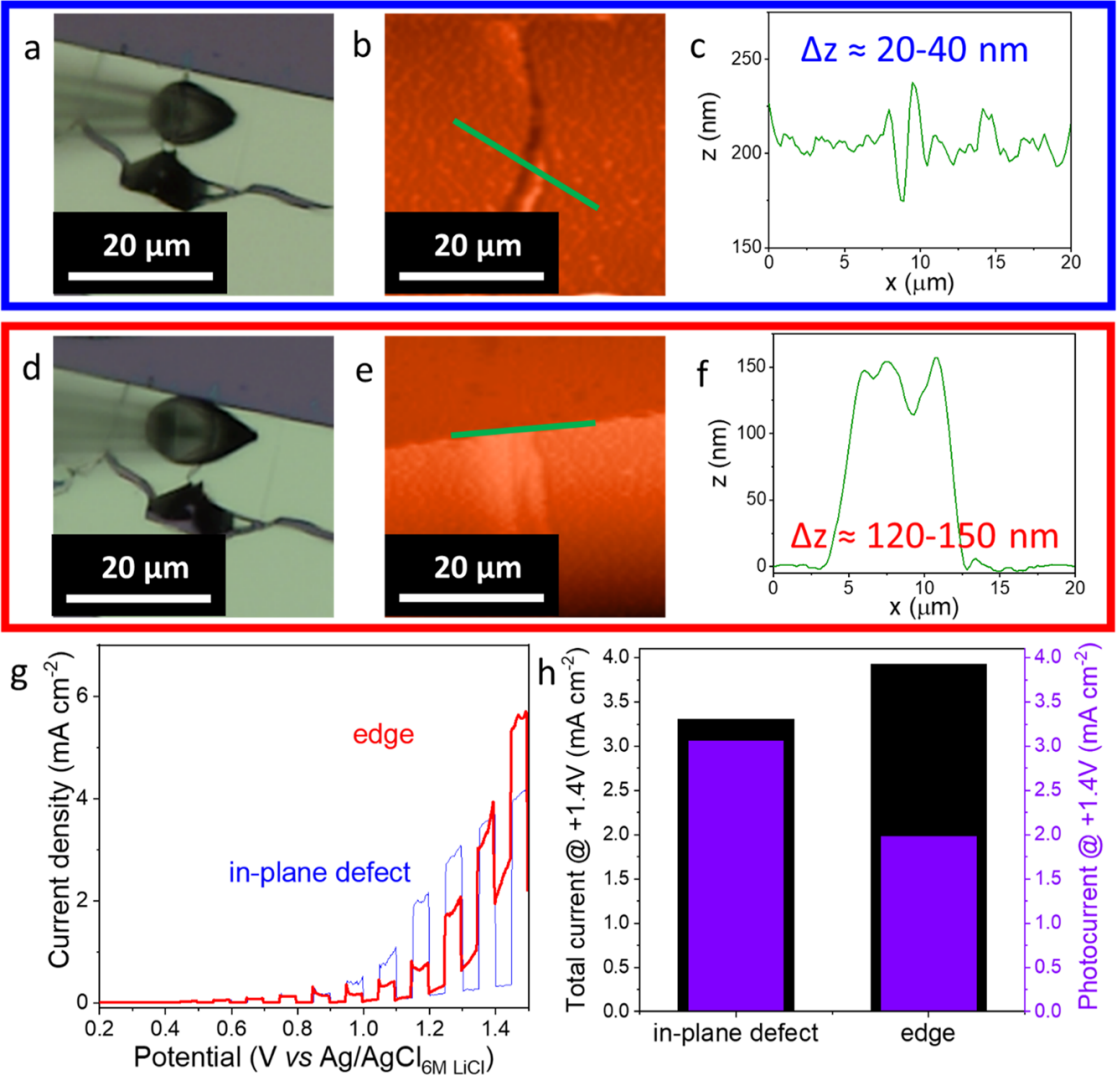
Optical (a, d) and AFM (b, e) micrographs of a MoSe_2_ bulk flake with deposited droplets on in-plane defects (a, b) and edges (d, e). The representative height profiles of in-plane defects (c) and edges (f) from cross sections (green lines marked in panels (b, e)). LSVs recorded for the illuminated droplets deposited on in-plane defects and edges (g); the sweep rate is 5 mV s^–1^. Total and photocurrent (data from panel (g)) bar diagrams plotted versus the kind of defect (h).

**Figure 5:**
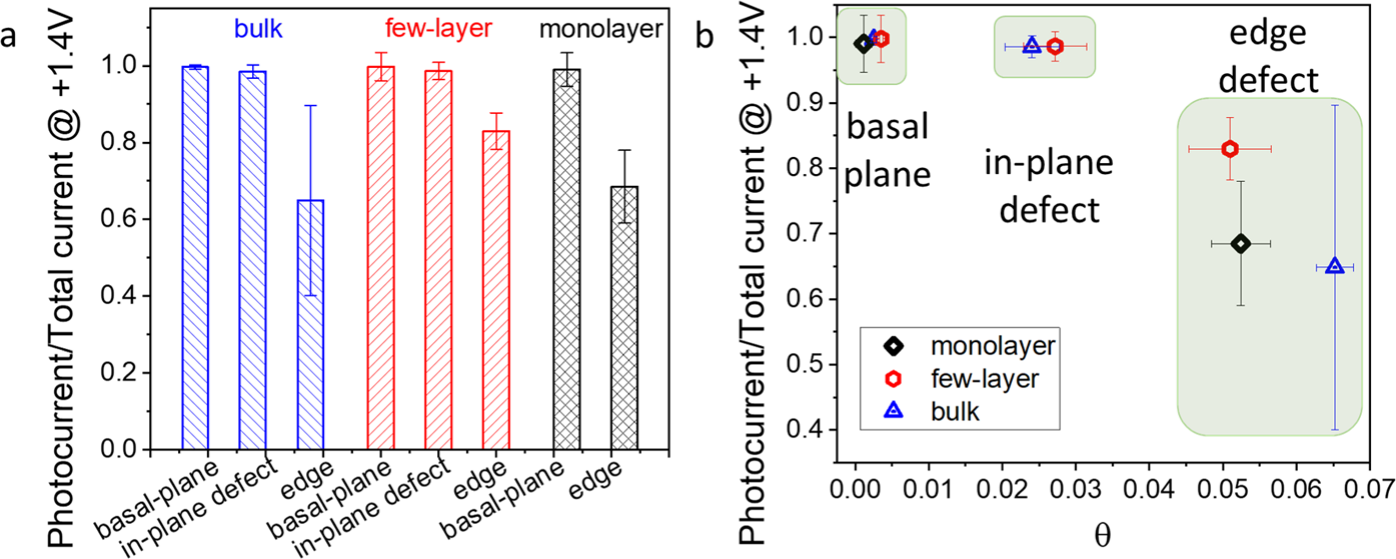
Effect of defect types on the PEC activity of layered MoSe_2_ specimens (a). Photocurrent/total current ratio values of monolayer, few-layer, and bulk MoSe_2_ flakes in the function of defect density and type (b).

Interestingly, 40 and 70% relative standard deviation accompanied the photocurrent measurements on the bulk materials (based on the analysis of the photovoltammetry curves), in the case of basal and in-plane defected samples, respectively (Figure S6a). To find the explanation for this large deviation, the PEC activity was analyzed as a function of different defects. In the case of bulk in-plane defects (deposited on the lower and upper positions on a terrace), the photovoltammetry curves were similar (Figure S8). On the contrary, the behavior of in-plane and edge sites on the bulk MoSe_2_ flake was different. Two droplets were deposited on the in-plane defect and edge (touching the peripheral part of the flake) (Figure 4a,d). AFM images (Figure 4b,e) and cross sections show the height profiles (Figure 4c,f) of these defects. The current densities for these structural domains are compared in Figure 4g, shown as a demonstrative example. The dark current on the edge defect sample increased notably, decreasing the photocurrent by 50%. On the contrary, in the case of in-plane defects, the dark current did not increase significantly (Figure 4h; more examples can be found in the Supporting Information, Figure S9). Additionally, a very similar trend was found for the few-layer and monolayer specimens (Figures S10 and S11). The results of the statistical analysis of all measurements regarding this phenomenon are depicted in Figure 5, where the studied structural parts distributed are as follows: 57 (in-plane defects), 32 (basal planes), and 11% (edge sites).

Figure 5a summarizes the photocurrent/total current ratio as the function of surface domains and the thickness of specimens. The basal plane behaves ideally for all layer thicknesses as all of the measured currents are photocurrents. If we introduce defects (in-plane ones) into basal planes, then the photocurrent ratio (photocurrent/total current) decreases only by 1–3%. Meanwhile, the edge defects cause 30–40% loss in the photocurrent ratio for all bulk, few, and monolayer specimens.

To provide a semiquantitative assessment on the effect of defect concentration, we borrowed a metric typically used in heterogeneous catalysis.^53^ The fraction of a heterogeneous surface covered by any additive or missing object (particles or vacancies, θ) describes the presence of different crystal faces, edges, imperfections, and impurities.^53^ We employ θ_defect_ to quantify the defect (in-plane or edge)-covered surface of an exfoliated flake. For mechanically exfoliated graphene, θ is close to zero for the perfect basal plane, while in the case of the defect-rich area (terrace in-plane defect), it was θ_edg_ = 0.053.^54^Figure 5b shows the relation between the number of defects and the photocurrent ratio of bulk, few, and monolayer MoSe_2_. In the case of the low θ_defect_ (almost smooth and clear surface), the ratio is almost 100% for all specimens. Introducing more defects, the defect density increases up to θ_defect_ = 0.02–0.03, and the photocurrent ratio decreases by 1–3%. Reaching the edges of the nanoflakes, the θ_defect_ value increases to 0.05–0.07; therefore, the photo/total current drops by 30–40%. Clearly, the increase of θ_defect_ influences the photo/total current ratio, reducing the PEC activity. Doubling the defect density (i.e., from 0.02–0.03 to 0.05–0.07) does not explain on its own the 10-fold increase (from 1–3 to 30–40%) in the photocurrent loss. This confirms that not only the defect density but also their nature (i.e., in-plane vs edge defects) is a decisive factor in dictating the photocurrent loss.

**Figure 6:**
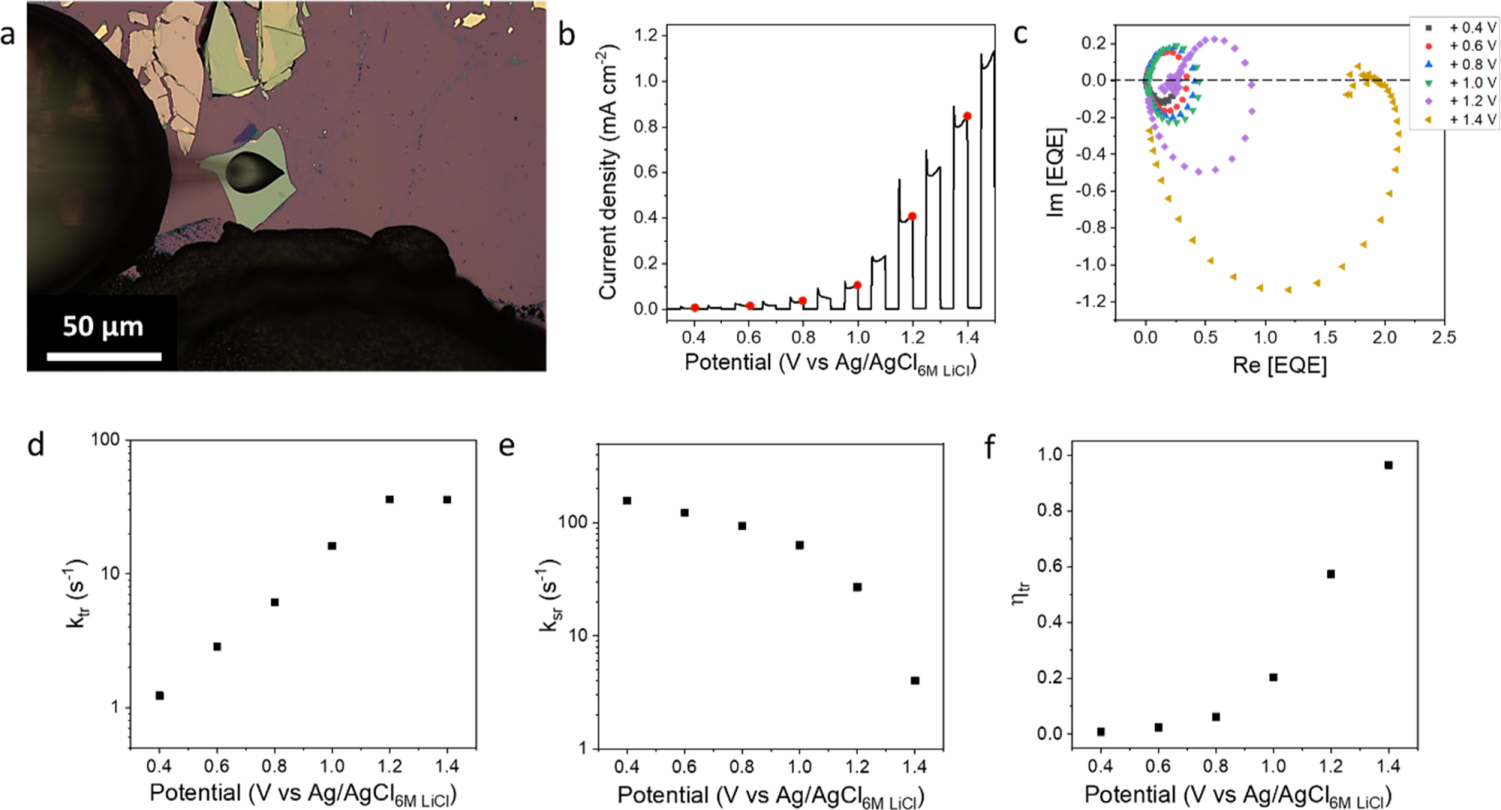
Optical micrograph of a MoSe_2_ bulk flake with deposited droplets on the basal plane (a). The thickness of this MoSe_2_ flake was 64.6 ± 0.7 nm. LSV recorded for the illuminated droplet (b); the sweep rate is 5 mV s^–1^. IMPS spectra (c) recorded for the illuminated droplet at the MoSe_2_ bulk flake at various applied potentials vs Ag/AgCl_6 M LiCl_ (indicated by red spots in panel (b)) under 35 mW cm^–2^ white light illumination (10% of it was applied as sinusoidal modulation). All measurements were applied in 6 M LiCl with 5 mM [Ru(NH_3_)_6_]^2+^. Charge transfer (d) and recombination (e) rate constants as the function of the applied potential determined from data presented in panel (c). Charge transfer efficiency (f) as the function of the applied potential, calculated from panels (d, e).

The charge carrier transfer and surface recombination characteristics of MoSe_2_ bulk specimens were further studied with IMPS.^55,56^ The optical micrograph for the MoSe_2_ bulk specimen is presented in Figure 6a (thickness is 64.6 ± 0.7 nm, see more in the Supporting Information, Figure S12). Figure 6b,c shows the photovoltammogram and IMPS spectra obtained in the deposited droplet on the bulk flake (potentials ranging from 0.4 to 1.4 V versus Ag/AgCl_6 M LiCl_). Almost perfect circles can be observed at the potentials from 0.4 to 1.0 V, meaning that the measured steady-state photocurrent is close to zero, and thus, surface recombination dominates the PEC behavior of the system. In the case of the IMPS spectrum, at 1.4 V, no semicircle can be identified in the upper quadrant, suggesting that the charge carrier transfer is the dominating process.^55^ The kinetic parameters were determined from the measured IMPS spectra and plotted versus the applied potential (Figure 6d,e). Rate constants, corresponding to charge carrier transfer (*k*_tr_) and surface recombination (*k*_sr_), were determined. An ascending and descending trend were found with increasing the potential for *k*_tr_ and *k*_sr_, respectively. While the *k*_tr_ increased linearly, the majority of the change in *k*_sr_ occurred at higher potential values. The relative charge transfer efficiency (η_tr_), presented as a function of applied potential (Figure 6f), was calculated from IMPS analysis (*k*_tr_/(*k*_tr_ + *k*_sr_)). The coupling of IMPS and our microdroplet microscopy approach opens a novel but challenging avenue for the mechanistic understanding of the structure-dependent PEC activity. Although we tried to record IMPS data for monolayer and few-layer samples as well, their smaller lateral size limited the photocurrents to a level that made the analysis unreliable.

Cyclic voltammograms of a model redox couple [Ru(NH_3_)_6_]^3+/2+^ were recorded in 6 M LiCl solution on MoSe_2_ (basal plane, in-plane defects, and edges of the bulk surface; Figure S13). The shape of CVs indicates activity differences for the various structural parts. The electrochemical activity of both defects (in-plane and edge) is different from that of the defect-free basal plane (Figure S13a). Another important observation was that, by decreasing the sample thickness, the shape of CVs changes (e.g., the separation of the oxidation and reduction peaks shrinks), indicating a gradually more reversible redox process.

**Figure 7:**
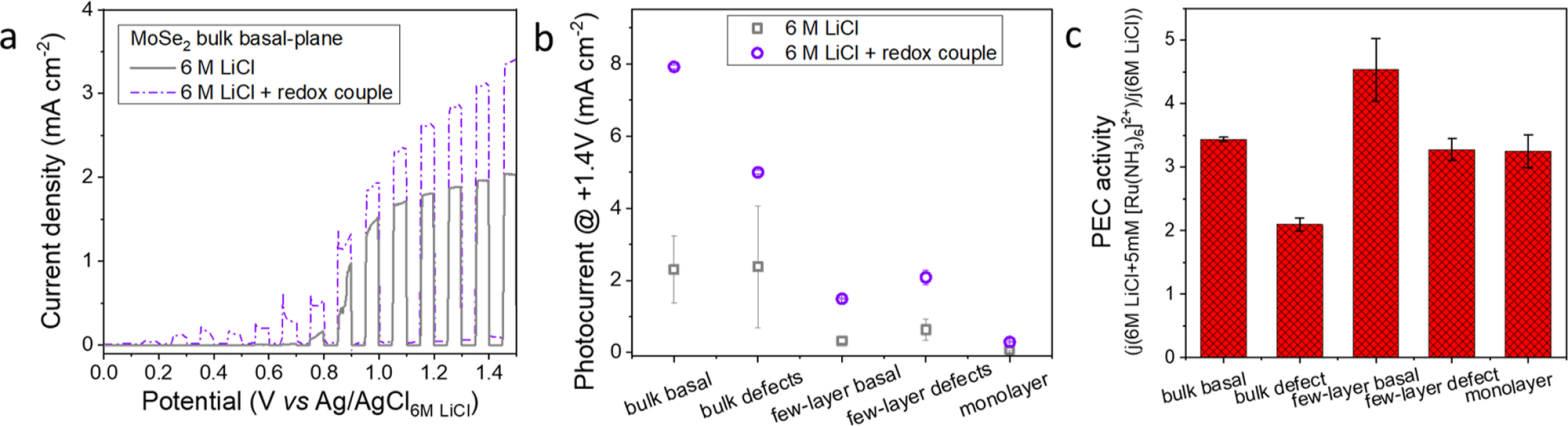
(a) LSVs recorded for the illuminated droplets deposited on bulk MoSe_2_ flakes, varying the composition of electrolytes; the sweep rate is 5 mV s^–1^. (b) Effect of thickness and defects on the photocurrents for MoSe_2_ flakes. (c) Correlation between the photocurrent densities recorded with and without the redox couple (6 M LiCl with 5 mM [Ru(NH_3_)_6_]^2+^ and 6 M LiCl).

The thickness of nanoflakes also affects the PEC activity (Figure 3), as reflected in the IPCE and APCE trends. The highest conversion efficiency was obtained for the bulk sample, while the monolayer achieved higher APCE values than the few-layer specimen. In the absence of any sacrificial electron donor (6 M LiCl solution), the low PEC activity was ascribed to the sluggish kinetics of water oxidation. Therefore, we examined how the sample thickness affects the photodriven electron transfer, if it is not limited by water oxidation kinetics, by applying a model redox couple ([Ru(NH_3_)_6_]^2+^). Figure 7a compares the photovoltammetry curves of water and [Ru(NH_3_)_6_]^2+^ oxidation on the MoSe_2_ bulk electrode. A less positive onset potential and higher photocurrents were registered in the case of [Ru(NH_3_)_6_]^2+^ due to the facile hole injection kinetics compared to the water oxidation. The maximum photocurrent values decrease with the introduction of defects and the thickness of nanosheets in both solutions (Figure 7b). The relative activity enhancement—the ratio of obtained photocurrents in 6 M LiCl with and without 5 mM [Ru(NH_3_)_6_]^2+^—is higher for the basal planes of both bulk and few-layer specimens than for the defected flakes (Figure 7c). The higher PEC activity measured for basal planes (bulk and few-layer) in the presence of sacrificial agent suggests the lack of water oxidation ability of these domains, while the lower ratio for the edge sites shows a better water oxidation performance. We have summarized all these trends in Table 1, comparing the PEC behavior of all the studied samples.

**Table 1:** Factors Underpinning the PEC Properties of 2D MoSe_2_ and WSe_2_ Nanoflakes

		light absorption range (eV)					
specimens	surface site	MoSe_2_	WSe_2_	light absorption	recombination	hole transfer to water	overall PEC performance
bulk	basal	1.42 ± 0.06	1.39 ± 0.08	sufficient	low	sluggish	good
	edge				high	very good	intermediate
few-layer	basal	1.62 ± 0.03	1.71 ± 0.08	intermediate	low	sluggish	intermediate
	edge				high	good	weak
monolayer	basal	1.71 ± 0.11	1.86 ± 0.08	insufficient	low	sluggish	weak
	edge				very high		weak

**Figure 8:**
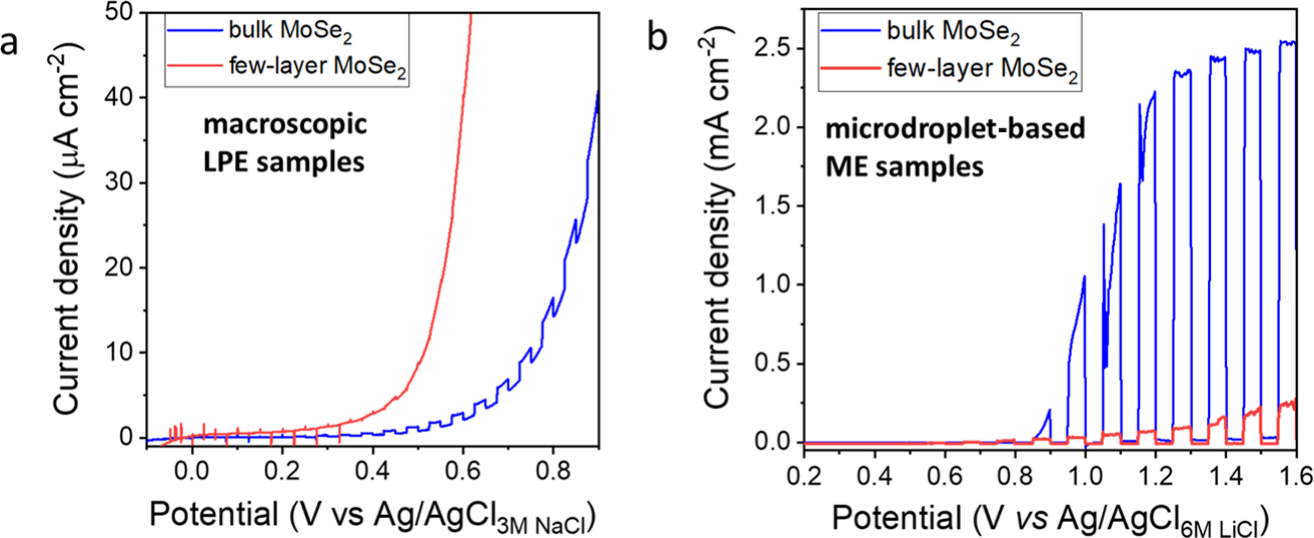
Comparison of PEC performance of liquid phase and micromechanically exfoliated samples. (a) LSVs recorded for the illuminated cells assembled with few-layer and bulk MoSe_2_/FTO electrodes; (b) microdroplets deposited on few-layer and bulk MoSe_2_ flakes. The sweep rate is 5 mV s^–1^, and LSVs are measured in 6 M LiCl solution.

An LPE process was employed to synthesize larger quantities of the nanoflakes, allowing the preparation of macroelectrodes. Using a centrifugation force-controlled separation, we obtained dispersions of bulk and few-layer flakes (see TEM images in Figure S14). After the bulk and few-layer films were deposited onto the FTO electrodes, the electrode coverage and the morphology of the flakes were analyzed (Figure S15a,b), and the absorption spectra of modified electrodes were recorded (Figure S15c, showing the characteristic absorption bands).^45−48^ Photovoltammetry profiles were recorded comparing the LPE (Figure 8a) and ME (Figure 8b) prepared flakes. The tests were run in 6 M LiCl solutions in both cases to compare only the role of different preparation methods. Only very small photocurrents (5–10 μA cm^–2^) were detected in the case of LPE prepared samples (Figure 8a), while in stark contrast, 1–2 orders of magnitude higher photocurrents (0.3–3 mA cm^–2^) were obtained for ME-based few-layer and bulk specimens applying microdroplet cells (Figure 8b). This difference agrees with the previously reported studies for LPE flake-based electrodes, where poor PEC behavior was detected without additional treatment (activation of active centrums or passivation of defects).^36,37^ The preparation of LPE flakes increases the defect density, and mostly, these defects refer to the increasing number of edges, rather than the presence of other structural defects (in-plane ones). The trend is also explained by the increasing number of flakes and their decreasing lateral size.^32^

## Conclusions

We studied the PEC activities of 2D MoSe_2_ and WSe_2_ flakes in the function of different structural domains (i.e., in-plane defects and edges). To explore the PEC performance of these structural parts, we applied our custom-developed microdroplet-based PEC microscope. We found that the edges are predominantly responsible for the decreased PEC activity, and the effect is more harmful for the thinner nanoflakes, compared to the bulk counterparts. Although defect sites have larger electrocatalytic activity (e.g., in water oxidation), the drastically increased charge carrier recombination is detrimental to the PEC performance. We tested the LPE produced MoSe_2_ and WSe_2_ bulk and few-layer flakes, achieving only a few μA cm^–2^ current densities because of the growing defect densities coming from the increased edge sites and decreased lateral size (area) of LPE flakes. In summary, our results suggest that the PEC activity of nanostructured 2D materials is inherently limited by the different requirements for a good charge carrier generator (such as a solar cell) and an electrocatalyst (see also Table 1), which poses limitations on the application of these materials in PEC solar energy conversion.

## Supporting Information Available

Experimental procedures with the details of the ME and LPE flake preparation, further details of our custom-made photoelectrochemical microscopy setup, investigated specimens and structural domains, the effect of in-plane defect vs edges, further photovoltammetry study of MoSe_2_ and WSe_2_, electron transfer analysis with additional IMPS results, and electron microscopic characterization of LPE flakes and modified FTO electrodes (PDF)
