# Supplementary material for: Structural Features Dictate the Photoelectrochemical Activities of Two-Dimensional MoSe2 and WSe2 Nanostructures
Source: J Phys Chem C Nanomater Interfaces. 2021 Mar 31;125(14):7701–10. doi: 10.1021/acs.jpcc.1c01265 (PMC8054242; doi:10.1021/acs.jpcc.1c01265)
Supplement: Supplementary file 1 — jp1c01265_si_001.pdf [file jp1c01265_si_001.pdf]

Supporting Information

Structural Features Dictate the Photoelectrochemical Activity of Two-dimensional $MoSe_2$ and WSe_2 Nanostructures

Péter S. Tóth[1,2*], Gábor Szabó[2], Csaba Janáky[2*]

[1]MTA Premium Post Doctorate Research Program, University of Szeged, Szeged, Hungary

[2]Department of Physical Chemistry and Materials Science, Interdisciplinary Excellence Center, University of Szeged, Szeged, Hungary

Corresponding Author

* To whom correspondence should be addressed.

Péter S. Tóth, Csaba Janáky

Tel: +36-62-544212, +36-62-546393

e-mail: toth.peter.sandor@chem.u-szeged.hu, janaky@chem.u-szeged.hu

Supporting Information Abstract

The additional results and discussion presented in the Supporting Information include the experimental procedures with the details of the ME and LPE flakes preparation; further details of our custom-made photoelectrochemical microscopy setup; investigated specimens and structural domains; effect of in-plane defect vs. edges; further photovoltammetry study of $MoSe_2$ and WSe_2; electron transfer analysis with additional IMPS results; and electron microscopic characterization of LPE flakes and modified FTO electrodes.

Mechanical exfoliation

The SiO_2/Si substrates were degreased by consecutive sonication in acetone and IPA for 5–5 minutes and cleaned using oxygen plasma (Harrick, PDC-32G). The crystals were repeatedly cleaved using scotch tape (Scotch Magic) to obtain a flat and pristine surface, which was pressed onto clean SiO_2/Si wafers. This was baked on a hot plate (CAT Scientific, MCS78) at 100 °C for 3–5 minutes. A fresh flake surface was exposed by peeling off scotch tape right after taking off the sample from hot plate. Suitable flakes were selected using optical microscopy and the wafer was immobilized on a microscope slide.

Liquid phase exfoliation

The LPE flakes were prepared using a bath sonicator operating at 37 kHz and 100% power for 12 h while chilled to maintain a stable temperature at 25 °C with a recirculating cooler system (J. P. Selecta, Digiterm TFT). The initial concentration was 1 mg mL^{-1} in water:IPA 3:1 mixture[1]. Subsequently, the dispersions were centrifuged at 200 g to remove any non-exfoliated material, the supernatant was then collected by pipetting and the centrifugation steps repeated to ensure a narrow distribution of flake lateral size and thickness. This sediment phase was called as bulk dispersion. For this cascade centrifugation process,[2] we collected and processed the supernatant for all cases. We started with a higher speed (1000g) and the supernatant was collected again, while the sediment was kept as well. Afterward, we centrifuged the supernatant, and sediment dispersions again using 2000g. Subsequently, this supernatant was kept, named as few-layer dispersion. Every centrifugation step was applied for 30 min at 15 °C. The resultant $MoSe_2$ and WSe_2 dispersions were stable in the water:IPA solution for several months with no detectable sedimentation.

The micro-droplet photoelectrochemical system

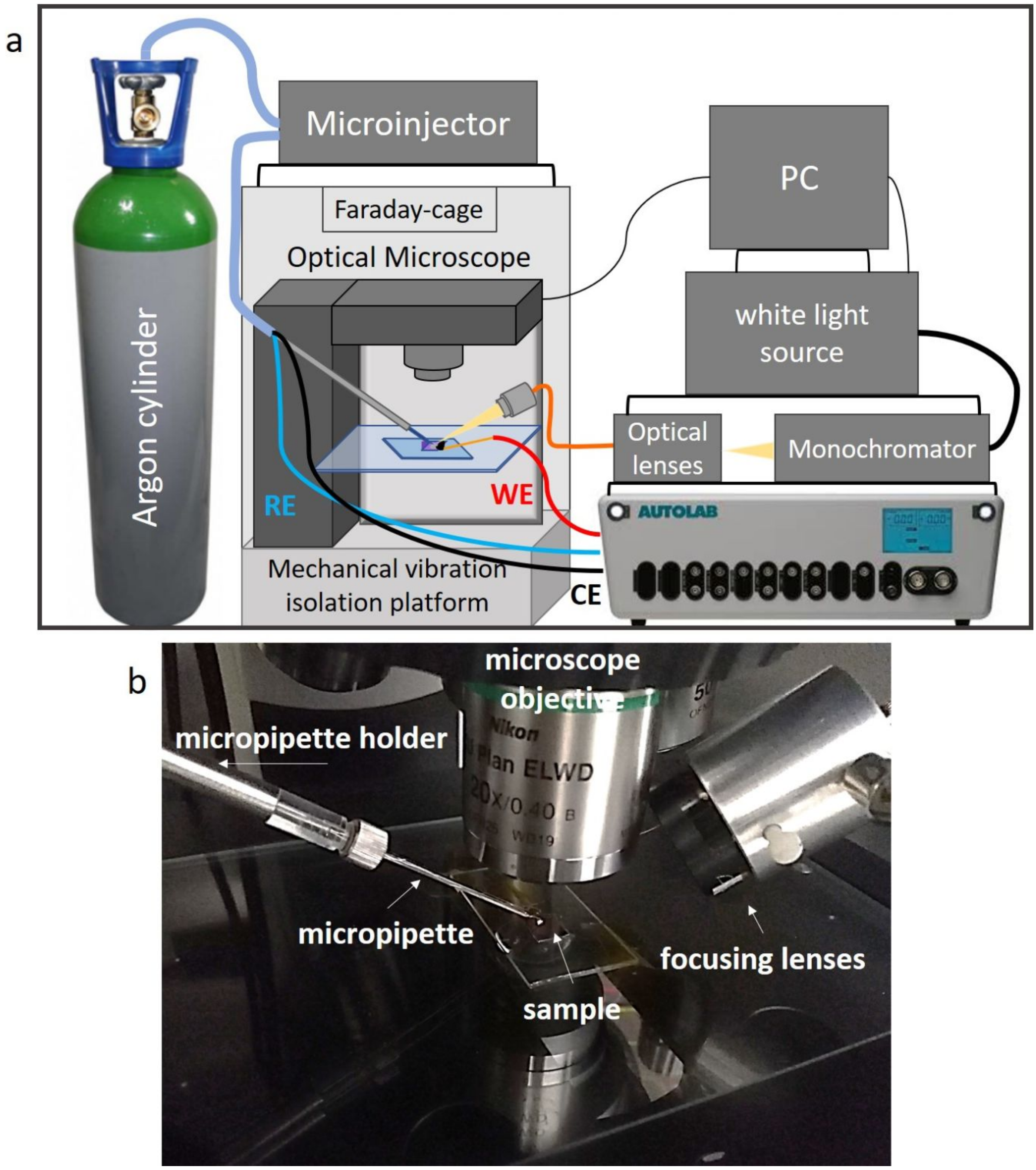

Figure S1. Experimental photoelectrochemical setup. (a) Scheme of experimental approach, (b) photograph of the sample area under the microscope objective.

All electrochemical measurements were performed in the deposited droplets of 6M LiCl aqueous electrolyte on the specimen surface, controlled by the Autolab potentiostat (Figure S1a). A micropipette puller was used to prepare the micropipette tip from a borosilicate

capillary (o.d. 1.5 mm, i.d. 1.1 mm). This tip was held by a motorized manipulator and connected to the micro-injector and inserted with the Pt counter, and Ag/AgCl pseudoreference electrodes (previously anodized in 0.1 M KCl solution). Droplet deposition, area measurements, and monitoring its shape change during EC/PEC experiments were recorded using an optical microscope equipped with a camera. Both white and monochromated illumination was conducted to the specimen using two lenses (f = 80mm, f = 30mm) to focus light on the fiber optical cable (50 μm core diameter) and onto the sample area (f = 70mm, f = 21mm) embedded in an aluminum tube (Figure S1b).

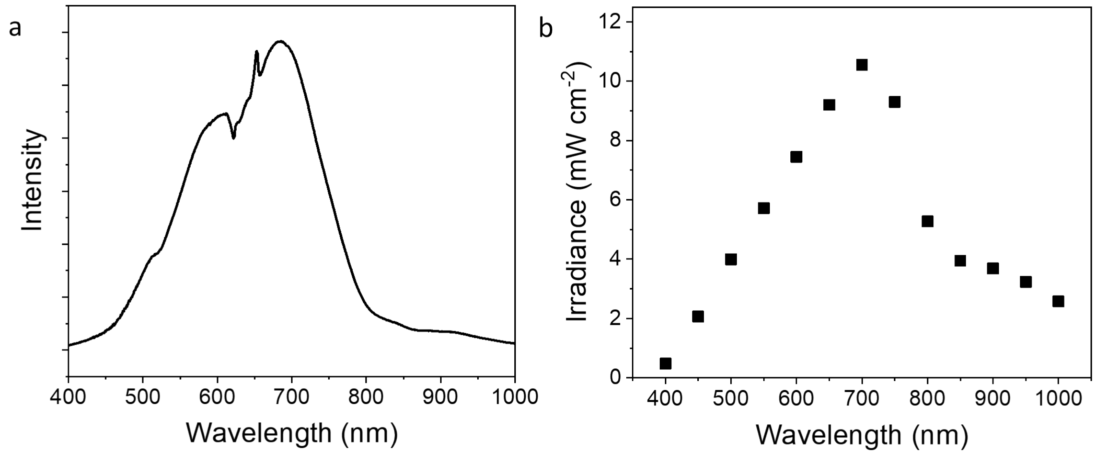

Figure S2. External illumination used for photoelectrochemical microscopy. (a) Absorption spectrum of white light. (b) Measured irradiance of monochromated spots at different wavelengths.

Characterization of samples

Atomic force micrograph and height profile from cross section (along the marked dashed black line in Figure S3a) of the $MoSe_2$ flakes and defects are presented in Figure S3a-b. In the case of few-layer $MoSe_2$ sample, we found a B^1_{2g} (at 357.5 cm^{-1}) inactive out-of-plane Raman mode[3], which helps for identification (Figure S3c). The ratio of the intensities of A_{1g} band (240.5–243 cm^{-1}) and Si band (520 cm^{-1}) are shown in Figure 3d.

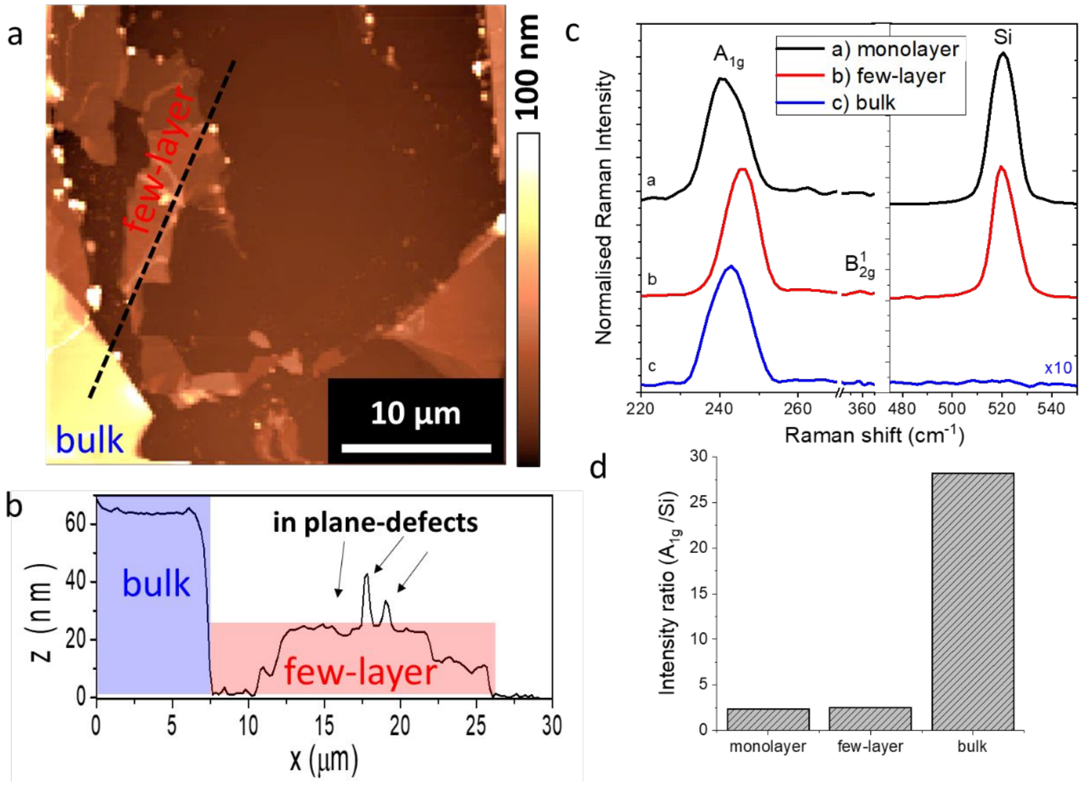

Figure S3. Morphological and structural characterization of layered $MoSe_2$ samples. (a) AFM micrograph of a selected few-layer/bulk flake region. (b) The representative height profile from cross sections (dashed black line marked in a). (c) Raman spectra of mono-, few-layer, and bulk flakes. (c) AFM micrograph of a selected few-layer/bulk flake region. (d) Ratio of the intensities of A_{1g} band and Si Raman band.

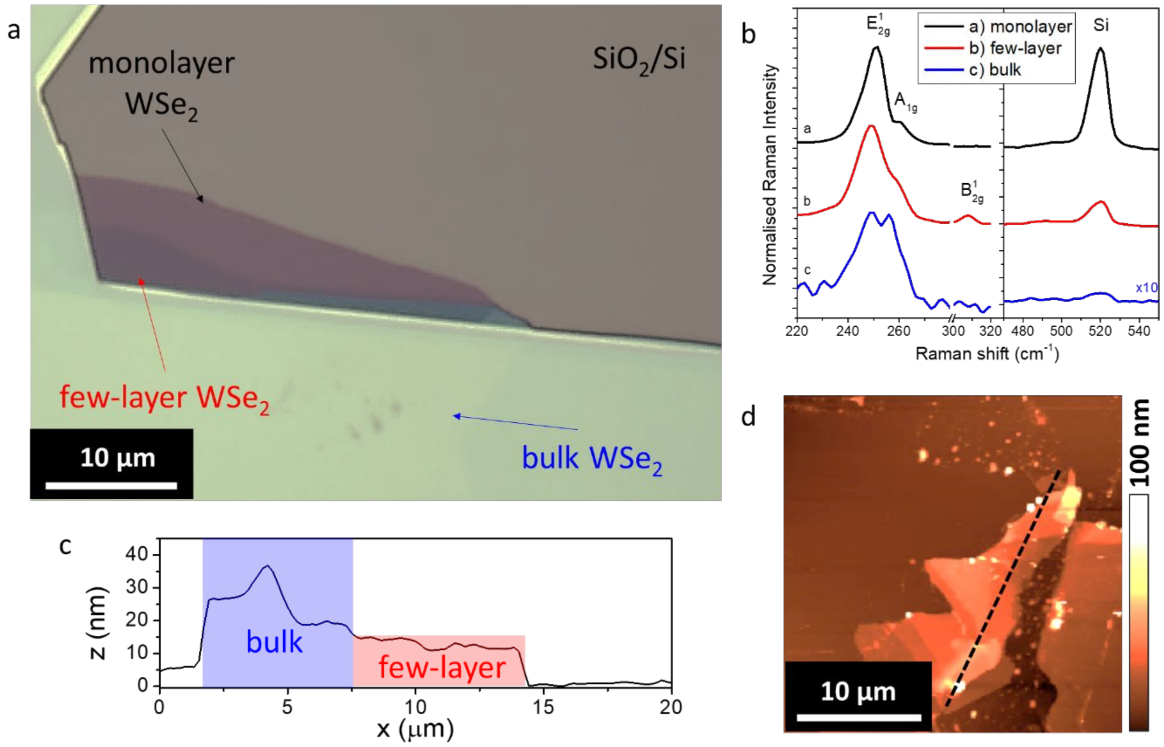

Figure S4. Morphological and structural characterization of layered WSe_2 samples. (a) Optical micrograph, (b) Raman spectra, (c) representative height profile from cross sections (dashed line marked in d), (d) AFM image of selected few-layer, and bulk WSe_2 flakes.

The optical micrograph in Figure S4a shows examples for mono-, few-layer and bulk WSe_2 flakes. For bulk WSe_2, two Raman signals were found at 248.5 cm^{-1} and 256.0 cm^{-1} (Figure S4b), predicting both the E^1_{2g} and the A_{1g} modes.[3,4] In the case of few-layer WSe_2 the E^1_{2g} and the normally inactive B_{2g} modes depicted. The position of E^1_{2g} mode changed with the number of layers, shifting to 251.5 cm^{-1} for monolayer WSe_2 flake. The AFM micrograph and height profile from cross section (along the marked dashed line in Figure S4d) of the WSe_2 flakes are presented in Figure S4c-d.

Figure S5 depicts representative examples for $MoSe_2$ and WSe_2 basal-plane and in-plane parts with defects (for example, terraces) on the selected flakes. These are also shown in Figure 1 (main text) on the example of $MoSe_2$ and WSe_2 flakes.

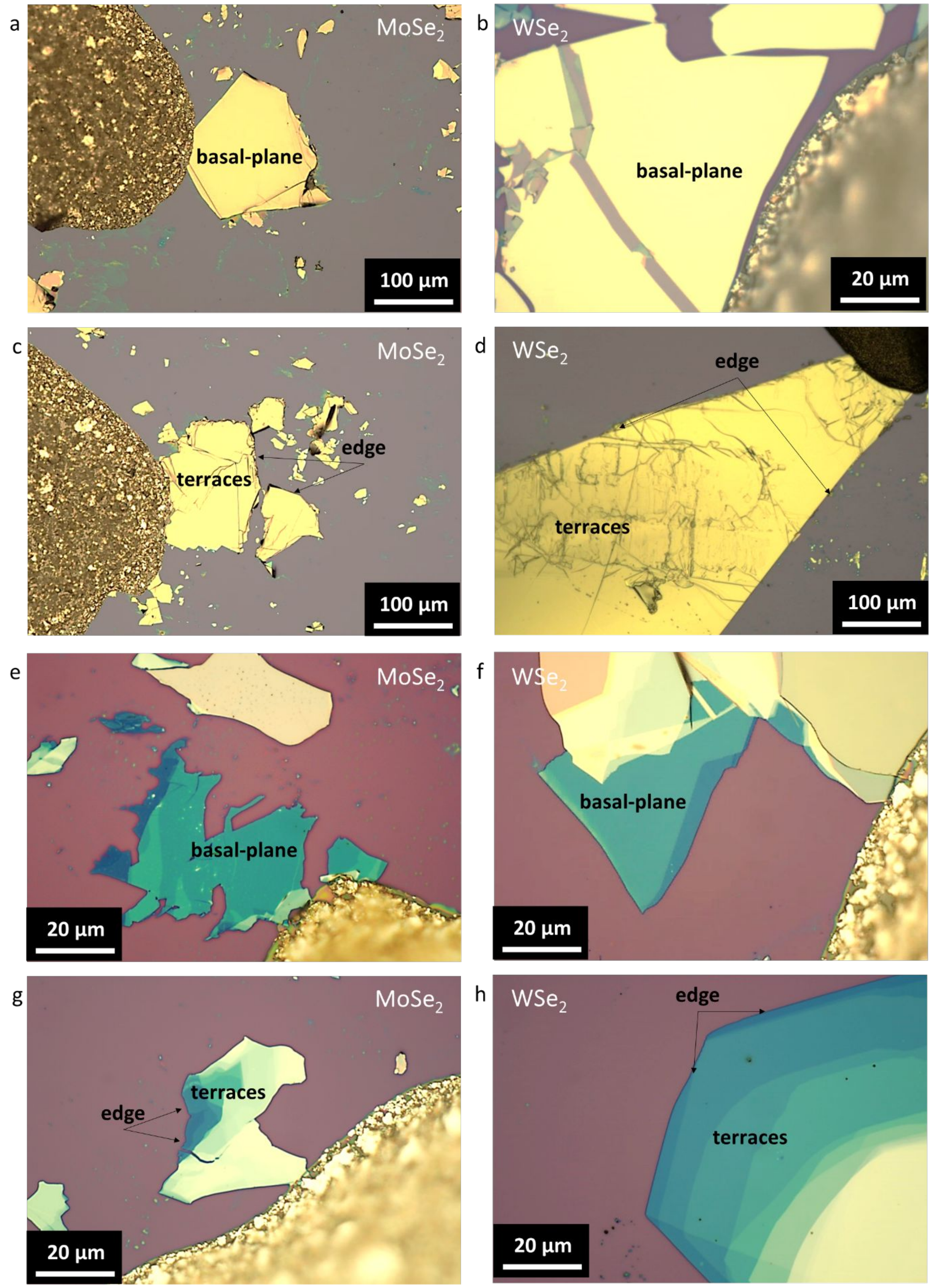

Figure S5. Optical micrographs of bulk (a-d) and few-layer (e-h) flakes of $MoSe_2$ (a, c, e, g) and WSe_2 (b, d, f, h), selecting basal-planes (a, b, e, f), and in-plane defects (c, d, g, h).

PEC activity study

The obtained photocurrent values were plotted as a function of sample thickness (bulk, few-layer, monolayer) and defect density for both $MoSe_2$ and WSe_2 (Figure S6a). It shows a decreasing trend upon decreasing the layer thickness, according the light absorption within layers[5]. The LSVs of mono-, few-layer, and bulk WSe_2 samples presented on Figure S6b.

Figure S7 shows the optical micrographs (Fig. S7a-d), the selected few-layer and bulk flakes region used as selected examples for the IPCE to APCE calculation. The additional AFM micrograph (Fig. S7b), and height profiles (Fig. S7c-e) are presented to demonstrate the exact layer thicknesses.

The PEC performance as a function of sample thickness and different structural domains were studied. The optical micrographs, the LSV curves and the total/photocurrents are presented (Figure S8-11), demonstrating the PEC behaviors of in-plane defects (Figure S8), of basal-planes vs. edge sites in the case of bulk sample (Figure S9) and for few- (Figure S10), and monolayers (Figure S11).

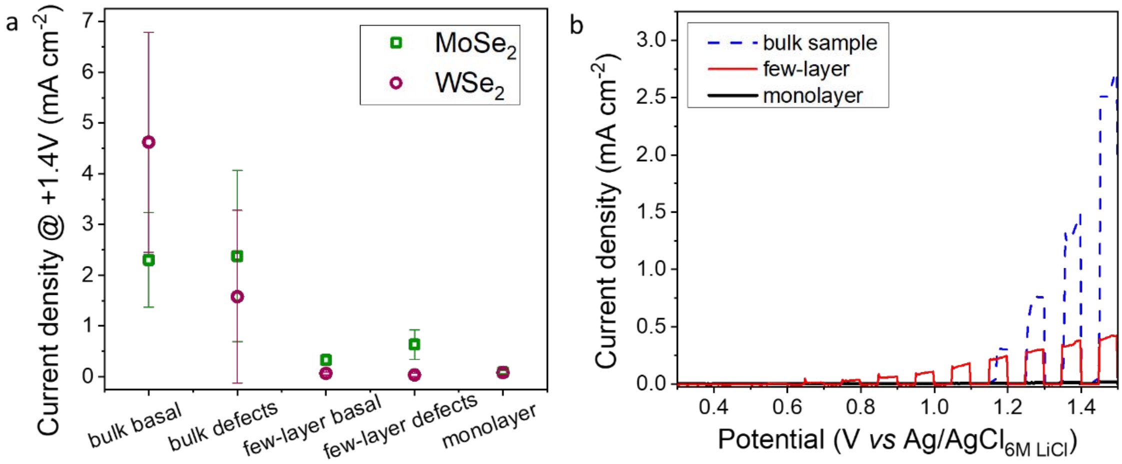

Figure S6. (a) Comparison of achieved currents in the function of number of layers and defects for $MoSe_2$ and WSe_2 flakes. (b) Photoelectrochemical behavior of WSe_2 layered samples, LSVs depending by the number of layers.

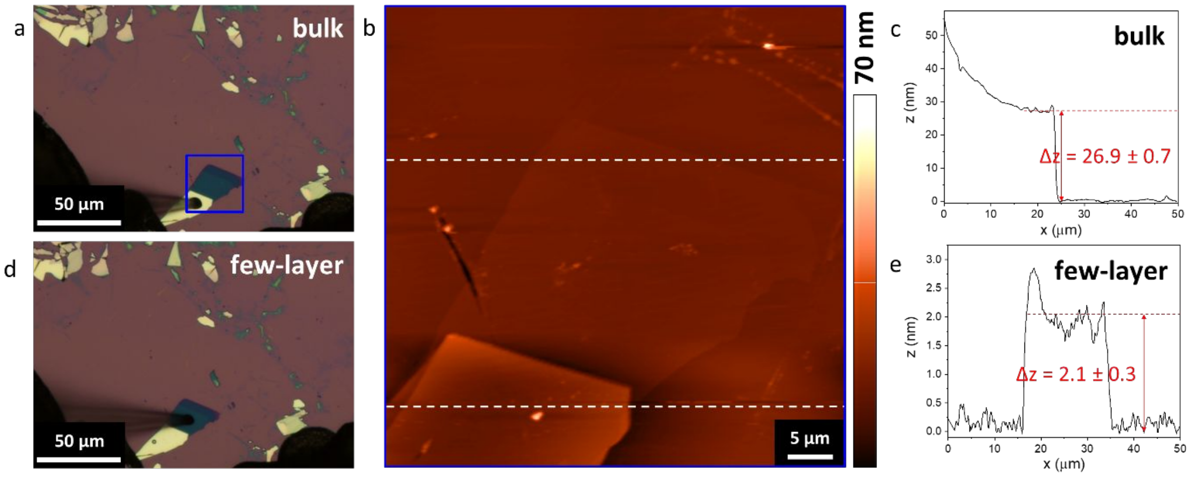

Figure S7. Optical micrographs of $MoSe_2$ bulk and few-layer flakes with deposited droplets on basal-planes (a, d). AFM image (b) of the selected bulk and few-layer flake region indicated by blue square in (a). The height profiles of the bulk and few-layer flakes' regions highlighted by the dashed white lines in (b). The thickness values are 26.9 ± 0.7 nm (c) and, 2.1 ± 0.3 nm (e) representing the thickness of bulk and few-layer flakes specimens, respectively.

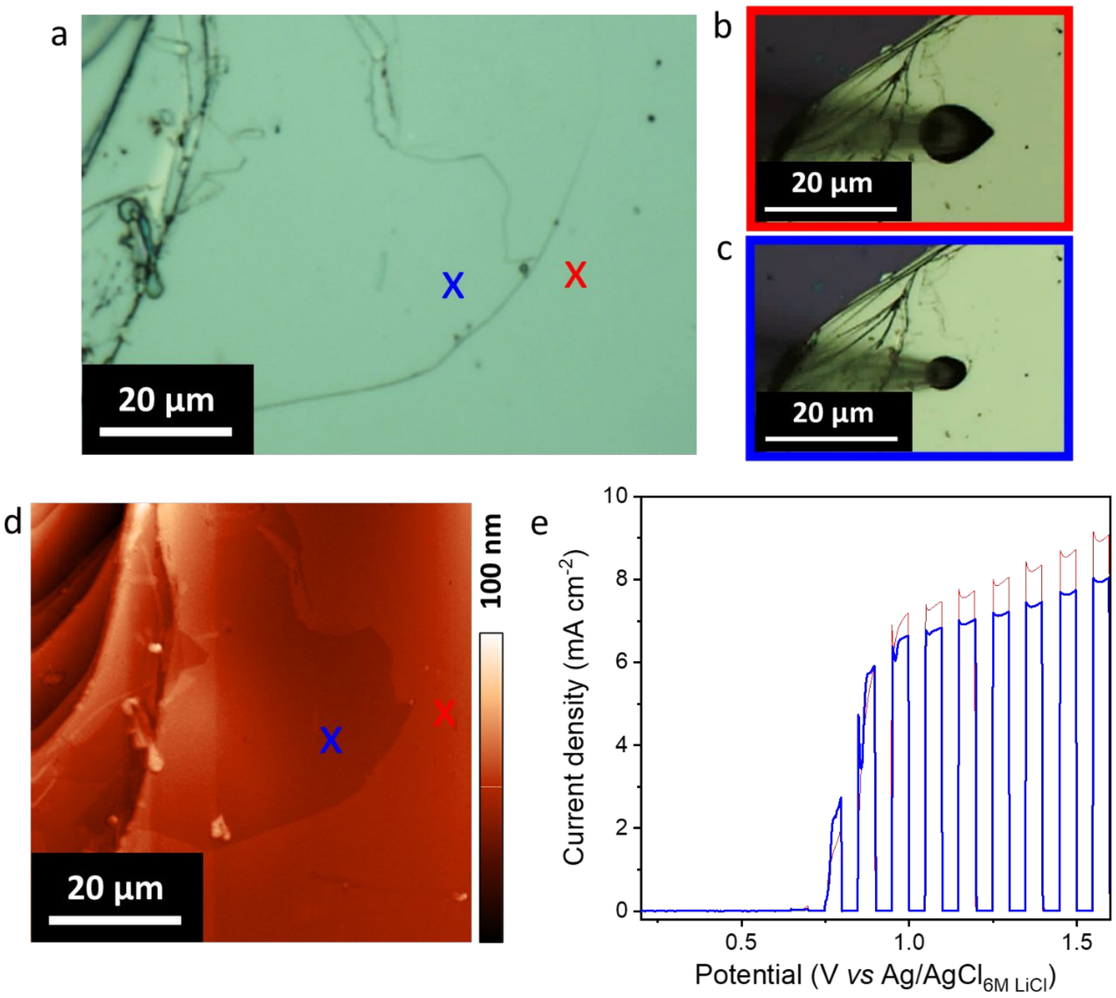

Figure S8. Optical micrographs of a $MoSe_2$ bulk flake (a), with deposited droplets on different parts of a terrace (b-c), these parts are marked by colored crosses. AFM micrograph (d) of the selected defected bulk flake region from micrograph (a) to identify the height differences between the two parts marked by colored crosses in (a). LSVs recorded for the illuminated droplets deposited on this terrace defect, sweep rate is 5 mV s^{-1}.

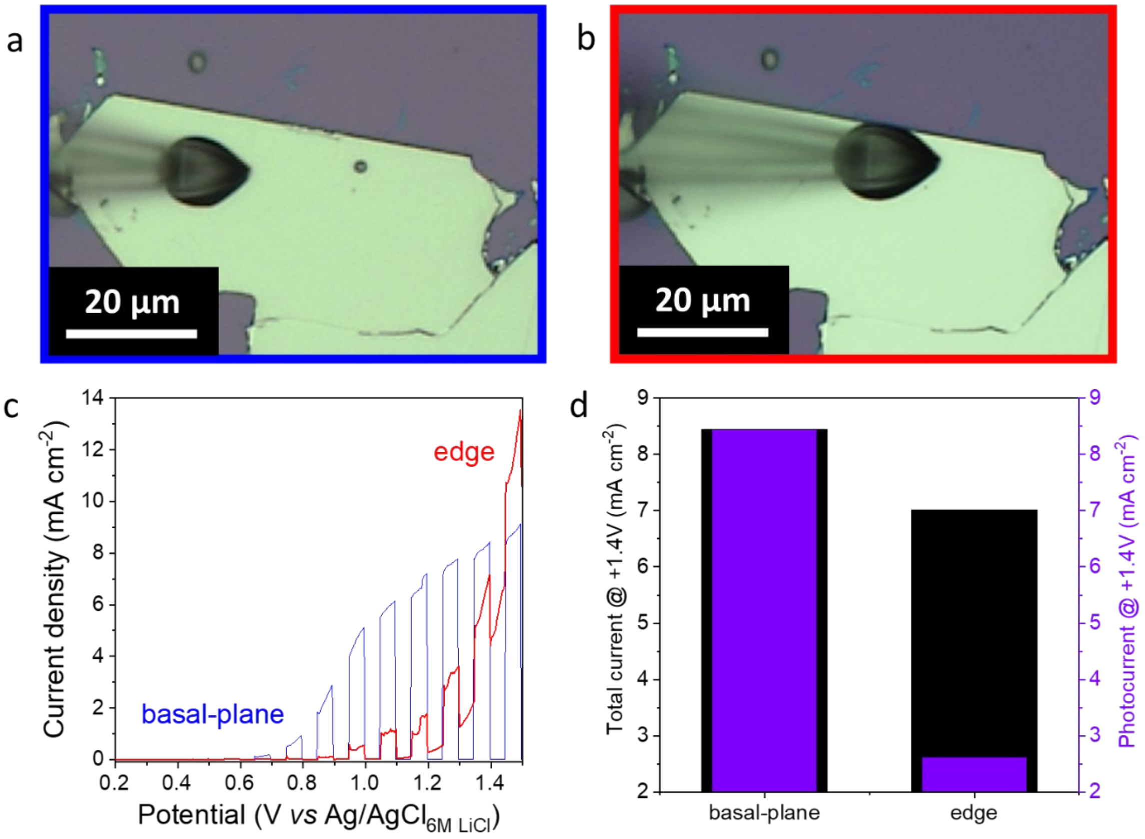

Figure S9. Optical micrographs of an $MoSe_2$ bulk flake with deposited droplets on basal-plane (a) and edge sites (b). LSVs recorded for the illuminated droplets deposited on basal-plane and edge, sweep rate is 5 mV s^{-1}. Total and photocurrents bar diagram plotted versus the kind of structural domains (d).

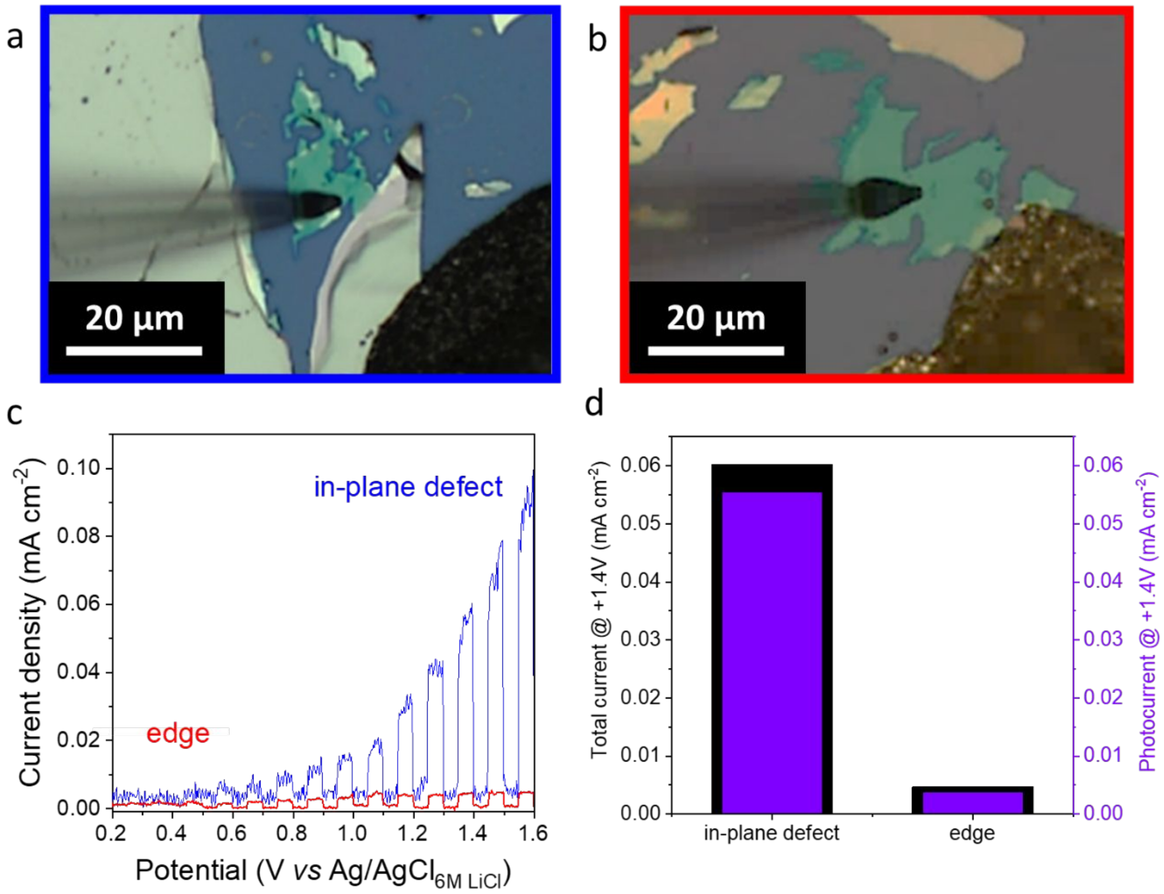

Figure S10. Optical micrographs of $MoSe_2$ few-layer flakes with deposited droplets on in-plane (a) and edge sites (b). LSVs recorded for the illuminated droplets deposited on in-plane and edges, sweep rate is 5 mV s^{-1}. Total and photocurrents bar diagram plotted versus the kind of defect (d).

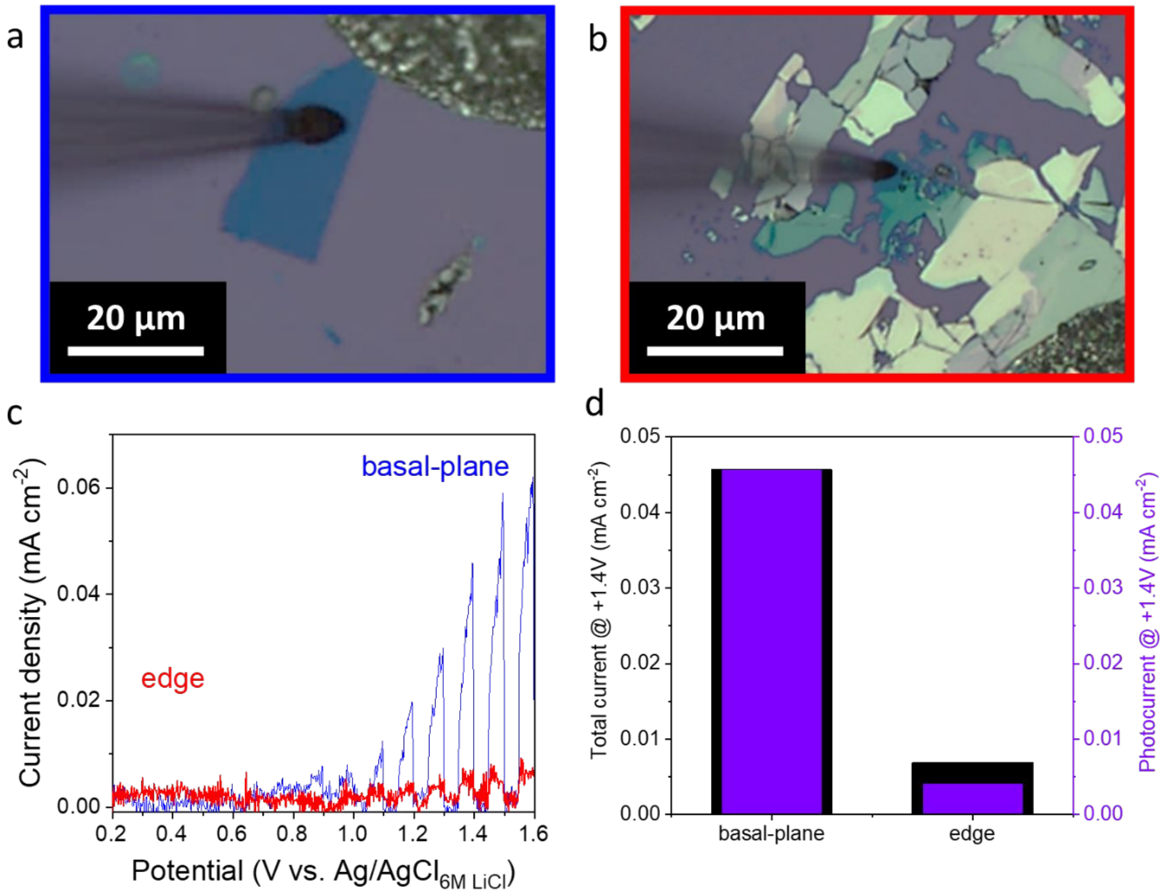

Figure S11. Optical micrographs of an $MoSe_2$ mono-layer flake with deposited droplets on basal-plane (a) and edge-plane (b). (c) LSVs recorded for the illuminated droplets deposited on basal-plane and edge, sweep rate is 5 mV s^{-1}. Total and photocurrents bar diagram plotted versus the kind of structural domains (d).

Analysis of electron transfer

The selected bulk flake for the IMPS analysis is shown by Figure S12, with the optical (Fig. S12a) and AFM micrographs (Fig. S12b), and height profile (Fig. S12c). Additionally, the Figure S13 shows the reduction/oxidation of $[Ru(NH_3)_6]^{3+/2+}$ in 6M LiCl applying cyclic voltammetry at $MoSe_2$ surface varying the sample thickness, and the defect density.

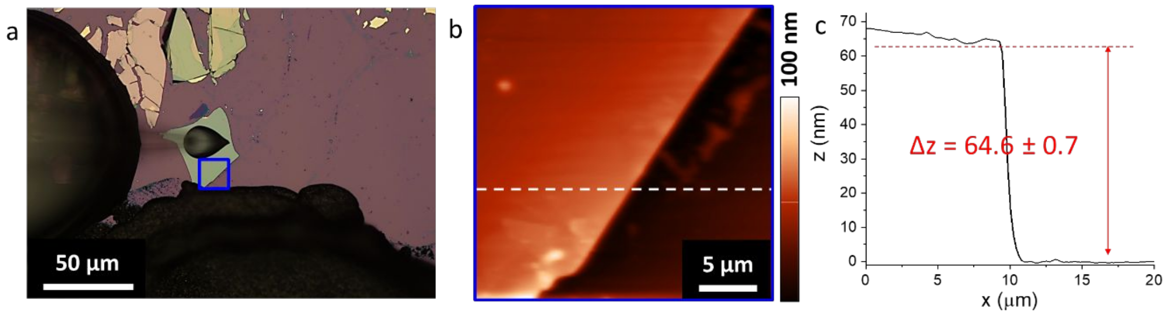

Figure S12. Optical micrograph of an $MoSe_2$ bulk flake with deposited droplet on basal-plane (a). AFM image (b) of the selected bulk flake region indicated by blue square in (a). The height profile of the bulk flake's region highlighted by the dashed white lines in (b), the thickness value is 64.6 ± 0.7 nm (a, b).

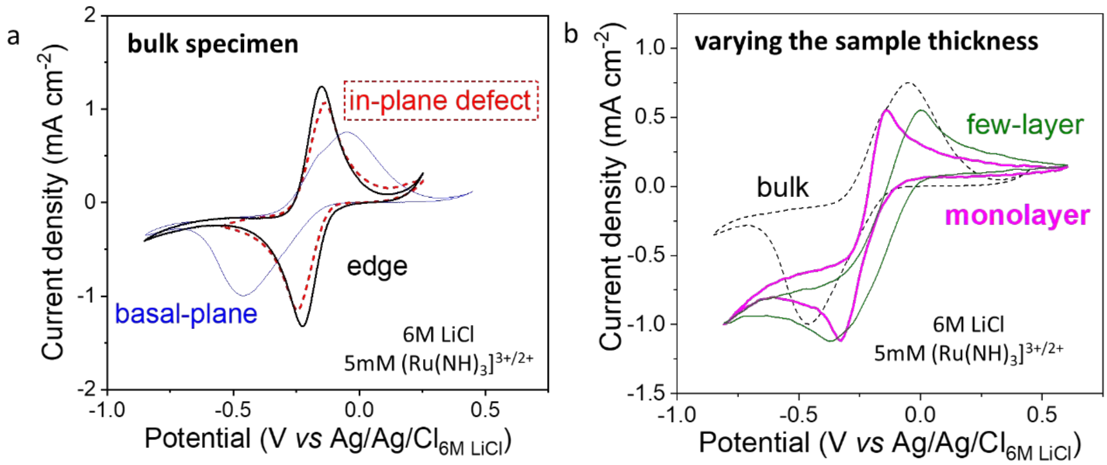

Figure S13. Electrochemical properties of $MoSe_2$ samples. (a) CVs of the illuminated droplets deposited on basal-plane, in-plane defect, and edge of bulk flakes. (b) CVs show that the EC behavior also depends on the thickness of specimens at basal-planes. The scan rate was 100 mV s^{-1} in 6M LiCl with 5 mM $[Ru(NH_3)_6]^{2+}$.

Morphological and optical characterization of LPE flakes and modified electrodes

Figure S14 shows the TEM images of $MoSe_2$ dispersions, as well as a statistical analyses to find the most probable size values. The log-normal distribution is characteristic of a random multiplicative process, e.g., ball milling, showing that exfoliation follows a linear fragmentation model, i.e., a process where the fragmentation is only driven by an external source, such as ultrasonic waves, therefore the statistical analyses are fitted using log-normal distributions.[6–8] The two samples, prepared by using different g-forces, have two different lateral size values. In particular, the 2000g (few-layer specimen) centrifugation processes give 35.5 ± 0.5 nm for lateral size. While, 265.4 ± 0.7 nm was found for lateral size of bulk $MoSe_2$ dispersion. The SEM images on Figure S15a-b presents the morphology of FTO deposited flakes of bulk (Figure S15a) and few-layer (Figure S15b) samples of $MoSe_2$. The absorption spectra of the bulk and few-layer $MoSe_2$ films on FTO electrodes prepared from dispersions are shown in Figure S15c.

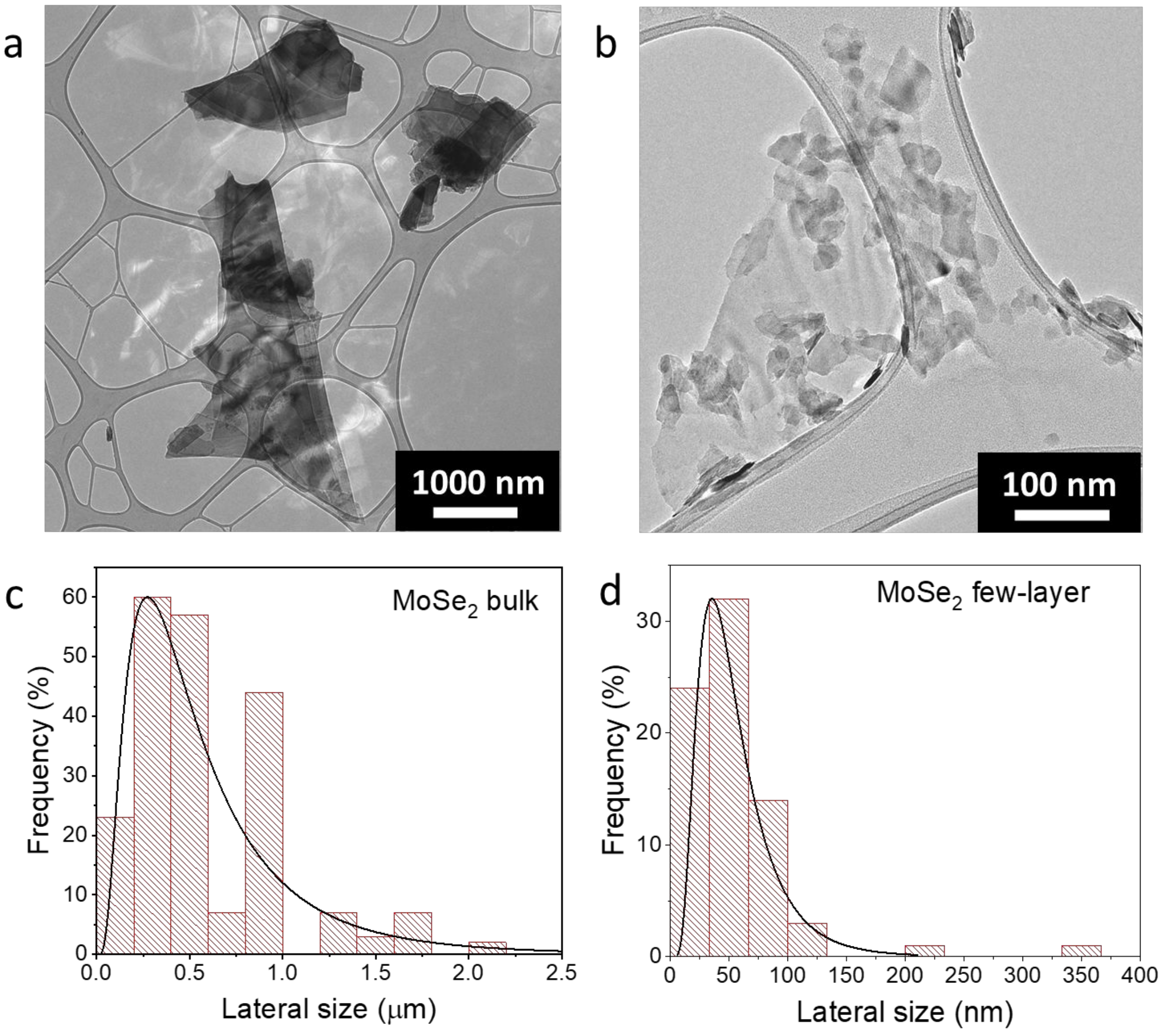

Figure S14. Morphological characterization of the $MoSe_2$ flakes. TEM images (a–b), and statistical analysis (c–d) on lateral size of selected flakes.

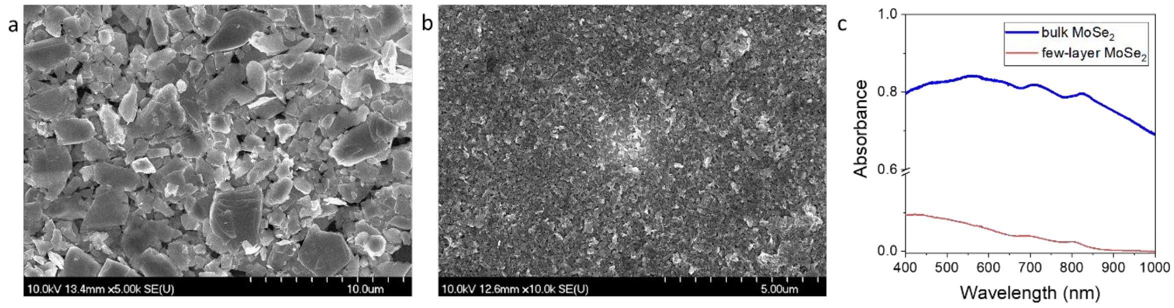

Figure S15. Morphological and optical characterization of the $MoSe_2$/FTO electrodes. SEM images of bulk (a) and few-layer (b) samples. (c) Vis-NIR absorption spectra of the LPE prepared $MoSe_2$ samples after film preparation from dispersions.

Supporting References

(1) Shen, J.; Wu, J.; Wang, M.; Dong, P.; Xu, J.; Li, X.; Zhang, X.; Yuan, J.; Wang, X.; Ye, M.; et al. Surface Tension Components Based Selection of Cosolvents for Efficient Liquid Phase Exfoliation of 2D Materials. *Small* **2016**, *12* (20), 2741–2749. https://doi.org/10.1002/smll.201503834.

(2) Backes, C.; Szydłowska, B. M.; Harvey, A.; Yuan, S.; Vega-Mayoral, V.; Davies, B. R.; Zhao, P.; Hanlon, D.; Santos, E. J. G.; Katsnelson, M. I.; et al. Production of Highly Monolayer Enriched Dispersions of Liquid-Exfoliated Nanosheets by Liquid Cascade Centrifugation. *ACS Nano* **2016**, *10* (1), 1589–1601. https://doi.org/10.1021/acsnano.5b07228.

(3) Tonndorf, P.; Schmidt, R.; Böttger, P.; Zhang, X.; Börner, J.; Liebig, A.; Albrecht, M.; Kloc, C.; Gordan, O.; Zahn, D. R. T.; et al. Photoluminescence Emission and Raman Response of Monolayer MoS_2, $MoSe_2$, and WSe_2. *Opt. Express* **2013**, *21* (4), 4908–4916. https://doi.org/10.1364/OE.21.004908.

(4) Ding, Y.; Wang, Y.; Ni, J.; Shi, L.; Shi, S.; Tang, W. First Principles Study of Structural, Vibrational and Electronic Properties of Graphene-like MX_2 (M=Mo, Nb, W, Ta; X=S, Se, Te) Monolayers. *Phys. B Condens. Matter* **2011**, *406* (11), 2254–2260. https://doi.org/https://doi.org/10.1016/j.physb.2011.03.044.

(5) Lewerenz, H. J.; Gerischer, H.; Lübke, M. Photoelectrochemistry of WSe_2 Electrodes: Comparison of Stepped and Smooth Surfaces. *J. Electrochem. Soc.* **1984**, *131* (1), 100–104. https://doi.org/10.1149/1.2115467.

(6) Cheng, Z.; Redner, S. Scaling Theory of Fragmentation. *Phys. Rev. Lett.* **1988**, *60* (24), 2450–2453. https://doi.org/10.1103/PhysRevLett.60.2450.

(7) Kouroupis-Agalou, K.; Liscio, A.; Treossi, E.; Ortolani, L.; Morandi, V.; Pugno, N. M.; Palermo, V. Fragmentation and Exfoliation of 2-Dimensional Materials: A Statistical Approach. *Nanoscale* **2014**, *6* (11), 5926–5933. https://doi.org/10.1039/C3NR06919B.

(8) Lago, E.; Toth, P. S.; Pugliese, G.; Pellegrini, V.; Bonaccorso, F. Solution Blending Preparation of Polycarbonate/Graphene Composite: Boosting the Mechanical and Electrical Properties. *RSC Adv.* **2016**, *6* (100), 97931–97940. https://doi.org/10.1039/c6ra21962d.
